# Phylogeny and Expansion of Serine/Threonine Kinases in Phagocytotic Bacteria in the Phylum *Planctomycetota*

**DOI:** 10.1093/gbe/evae068

**Published:** 2024-03-28

**Authors:** Anna Odelgard, Emil Hägglund, Lionel Guy, Siv G E Andersson

**Affiliations:** Molecular Evolution, Department of Cell and Molecular Biology, Science for Life Laboratory, Uppsala University, Uppsala, Sweden; Molecular Evolution, Department of Cell and Molecular Biology, Science for Life Laboratory, Uppsala University, Uppsala, Sweden; Department of Medical Biochemistry and Microbiology, Science for Life Laboratory, Uppsala University, Uppsala University, Uppsala, Sweden; Molecular Evolution, Department of Cell and Molecular Biology, Science for Life Laboratory, Uppsala University, Uppsala, Sweden

**Keywords:** phagocytosis, bacteria, “*Candidatus* Uabimicrobium amorphum”, signal transduction systems, protein kinases

## Abstract

The recently isolated bacterium “*Candidatus* Uabimicrobium amorphum” is the only known prokaryote that can engulf other bacterial cells. Its proteome contains a high fraction of proteins involved in signal transduction systems, which is a feature normally associated with multicellularity in eukaryotes. Here, we present a protein-based phylogeny which shows that “*Ca*. Uabimicrobium amorphum” represents an early diverging lineage that clusters with the *Saltatorellus* clade within the phylum *Planctomycetota*. A gene flux analysis indicated a gain of 126 protein families for signal transduction functions in “*Ca.* Uabimicrobium amorphum”, of which 66 families contained eukaryotic-like Serine/Threonine kinases with Pkinase domains. In total, we predicted 525 functional Serine/Threonine kinases in “*Ca.* Uabimicrobium amorphum”, which represent 8% of the proteome and is the highest fraction of Serine/Threonine kinases in a bacterial proteome. The majority of Serine/Threonine kinases in this species are membrane proteins and 30% contain long, tandem arrays of WD40 or TPR domains. The pKinase domain was predicted to be located in the cytoplasm, while the WD40 and TPR domains were predicted to be located in the periplasm. Such domain combinations were also identified in the Serine/Threonine kinases of other species in the *Planctomycetota*, although in much lower abundances. A phylogenetic analysis of the Serine/Threonine kinases in the *Planctomycetota* inferred from the Pkinase domain alone provided support for lineage-specific expansions of the Serine/Threonine kinases in “*Ca.* Uabimicrobium amorphum”. The results imply that expansions of eukaryotic-like signal transduction systems are not restricted to multicellular organisms, but have occurred in parallel in prokaryotes with predatory lifestyles and phagocytotic-like behaviors.

**Significance**

Expansions of signal transduction systems have been linked to multicellularity in eukaryotes, but such expansions are less common and have been less investigated in prokaryotes. We show that “*Candidatus* Uabimicrobium amorphum”, which is the only known bacterium that can internalize other bacteria via a phagocytosis-like process, has massively expanded its repertoire of Serine/Threonine kinases, many of which are membrane spanning and contain tandem arrays of WD40 or TPR domains. Although Serine/Threonine kinases as well as proteins with WD40 or TPR domains are broadly present in eukaryotes, different protein domain architectures suggest that these signal transduction systems have undergone lineage-specific expansion in “*Ca*. Uabimicrobium amorphum” independently of their expansions in the eukaryotes.

## Introduction

The divergence in structure between eukaryotic and prokaryotic cells has been said to represent the greatest evolutionary discontinuity (Stanier et al. 1963) and transition (Cavalier-Smith 2014) in the history of life. The eukaryotic cell is characterized by a complex intracellular membrane system, including a nuclear membrane and energy-producing organelles, while the prokaryotic cell is defined by the lack thereof. Over the years, many theories have been proposed for the origin of the eukaryotic cell (Poole and Gribaldo 2014; Eme et al. 2018; López-García and Moreira 2020; Gabaldón 2021) and these can roughly be divided into “mitochondria-early” (Martin and Müller 1998), “mitochondria-intermediate” (Vosseberg et al. 2021), and “mitochondria-late” (Cavalier-Smith 1987) hypotheses, depending on the time at which the mitochondrion originated in relation to the other internal membrane structures. Despite much controversy in the field, all theories assume that the origin of the eukaryotic cell was a rare event that has only happened once (Lane 2011).

Bacteria of the phylum *Planctomycetota* challenge this simplistic view of two contrasting cell architectures by containing complex intracellular structures not normally seen in prokaryotes (Fuerst 2005; Fuerst and Sagulenko 2011). Although the *Planctomycetota* cells have been unambiguously classified as bacteria by genetic analyses (Fuerst and Webb 1991; Lindsay et al. 2001; Jogler et al. 2011), they have complex intracellular membrane network systems formed by invaginations of the cytoplasmic membrane (Lindsay et al. 1997, 2001; Lee et al. 2009; Pinos et al. 2016). This suggests that the eukaryotic cell architecture may not be as unique as previously thought. By studying the *Planctomycetota* cells and their genomes, we may gain knowledge about the selective forces and the early steps involved in the transition towards increased cellular complexity.

The remarkable cellular features discovered in the PVC (*Planctomycetota*, *Verrumicrobiota*, and *Chlamydiota*) superphylum continue to surprise. As recently as 2019, several new isolates with novel traits not previously observed within the *Planctomycetota* phylum were described. Four of these novel isolates formed a monophyletic clade named *Saltatorellus* (Wiegand et al. 2019). These bacterial cells can change their shape, fuse together, and they are capable of switching between cell division by budding and binary fission. Both binary fission and budding are common traits in members of the *Planctomycetota*. However, no other species can switch between different means of cell proliferation. The *Saltatorellus* species possess a bacterial actin-like homolog called saltatorellin, which may be involved in the shape-shifting ability. Common to all *Saltatorellus* and *Planctomycetota* species is the absence of FtsZ, a protein otherwise essential for cell division in bacteria (Wiegand et al. 2020).

Even more astonishing was the discovery of “*Candidatus* Uabimicrobium amorphum”, the first bacterium shown to be able to ingest other bacterial cells and incorporate them into phagosome-like vacuoles (Shiratori et al. 2019). The sizes of the “*Ca.* Uabimicrobium amorphum” cells were estimated to be 4 to 5 µm in diameter, but cells as large as 10 µm were observed when other bacterial cells were present in the growth medium. The 9.5 Mb genome of “*Ca*. Uabimicrobium amorphum” is unusually large compared to other bacteria and was predicted to contain 6,600 genes (Shiratori et al. 2019). Even though several *Planctomycetota* species are able to ingest large macromolecules, phagocytosis—the ability to engulf other cells—has been considered a feature uniquely associated with eukaryotes. This discovery challenges the assumption that phagocytosis, which is a very energy-demanding process, could only have evolved in cells that contained a mitochondrion (Lane and Martin 2010; Martin et al. 2015). Extended intracellular membrane systems, as in the *Planctomycetota* cells, may suffice to provide the energy required for such a process. However, no homologs to eukaryotic genes involved in phagocytosis, except for an actin-like protein, could be detected in the genome of this bacterium, and the mechanisms involved remain elusive (Shiratori et al. 2019).

Comparative genomics studies of the *Planctomycetota* phylum have started to yield insights into the molecular events associated with the emergence of intracellular network structures, such as the remodeling of the otherwise universal peptidoglycan cell wall (Mahajan et al. 2020a) and an expansion of proteins involved in regulatory and signal transduction systems in the family *Gemmataceae* (Mahajan et al. 2020b). Interestingly, many of the expanded proteins in the *Gemmataceae* were found to have unique multidomain architectures, including domains commonly found in prokaryotes, such as the ECF sigma factor domain, combined with various types of repeat domains that are highly abundant in eukaryotes, such as the WD40 domain (Mahajan et al. 2020b). Among the expanded proteins, members of the *Gemmataceae* harbor circa 100 protein kinases with the Pkinase domain, which is the catalytic subunit that transfers the γ-phosphate from ATP to an amino acid of a target protein. Some of the protein kinases were confirmed to be expressed in *Tuwongella immobilis*, a member of the *Gemmataceae* (Seeger et al. 2021). The high number of kinases in the *Gemmataceae* is comparable to the abundance of kinases in eukaryotes with similar-sized proteomes (Mahajan et al. 2020b). Likewise, the genome sequencing and functional predictions of the 6,600 genes in the “*Ca.* Uabimicrobium amorphum” genome indicated an atypically large number of genes coding for proteins involved in signal transduction (Shiratori et al. 2019), although the nature of these proteins was not further investigated.

In eukaryotes, Ser/Thr/Tyr kinases represent the main signal transduction system. These kinases mediate the transfer of the γ-phosphate of ATP to Serine, Threonine, or Tyrosine on the target protein, which initiates a cascade of downstream regulatory events. The kinase domain is 250 to 300 amino acids and contains specific conserved motifs, including three key amino acids, lysine (K) in subdomain II and two aspartic acid residues in subdomains VIb (D1) and VII (D2), which are thought to be essential for the catalytic activity of the kinase (Hanks and Hunter 1995; Krupa and Srinivasan 2005; Pereira et al. 2011). Because of their universal occurrence in eukaryotes, these kinases have been referred to in the literature as “eukaryotic-like Serine/Threonine kinases”.

Bacteria rely mostly on two-component systems for signal transduction, such as histidine kinases and their associated response regulators. Kinases that phosphorylate tyrosine in bacteria are named BY-kinases (Bacterial tYrosine kinases), but these show no sequence similarity to the eukaryotic-like Serine/Threonine kinases (Grangeasse et al. 2012). Thus, tyrosine phosphorylation is performed by different enzymes in bacteria and eukaryotes, and the eukaryotic-like Serine/Threonine kinases were initially thought not to have homologs in prokaryotes. However, we now know that bacteria also contain kinases that are structurally similar to the Ser/Thr/Tyr kinases in eukaryotes, although they only phosphorylate serine and threonine residues in the target proteins (Krupa and Srinivasan 2005; Pereira et al. 2011). Overall, the Pkinase domain, which is the catalytic domain of the Ser/Thr/Tyr kinases in eukaryotes, was identified in 46% of 4,620 bacterial proteomes (Mahajan et al. 2020b). Some of these kinases are membrane proteins, while others are cytoplasmic, and the enzymes in both bacteria and eukaryotes can contain additional domains that either mediate binding to ligands or other proteins. The bacterial Ser/Thr kinases and their associated Ser/Thr phosphatases have been implicated in the regulation of a variety of physiological processes, such as cell division and cell wall biosynthesis, but they may also be involved in stress response, biofilm formation, and virulence (Janczarek et al. 2018). Throughout this manuscript, we refer to the eukaryotic kinases as Ser/Thr/Tyr kinases (STYKs) and their homologs in bacteria as Ser/Thr kinases (STKs).

Proteins with the Pkinase domain in bacteria, archaea, and eukaryotes were probably present already in the Last Universal Common Ancestor (LUCA), and the domain may thus share a common evolutionary origin (Grangeasse et al. 2012; Stancik et al. 2018). However, unlike their high abundances in the eukaryotes, only a few bacterial species contain more than 15 STKs per proteome, including members of the *Planctomycetota*, *Myxobacteria*, *Cyanobacteria*, hyphal and mycelial *Actinobacteriai* and filamentous *Chloroflexi* (Pérez et al. 2008). Until now, the highest relative fraction of STKs in prokaryotes had been found in *Myxobacteria* (Pérez et al. 2008). A phylogeny based on the Pkinase domains in these bacteria suggested extensive gene duplications, most of which were species or strain-specific (Pérez et al. 2008). Some of the duplicated genes were located in immediate proximity to each other, indicative of tandem duplications. In the myxobacteria, the number of STKs has been shown to increase exponentially with genome size, from 14 genes in the 5 Mb genome of *Anaeromyxobacter* to 99 genes in the 9.1 Mb genome of *Myxococcus xanthus* and to as many as 317 to 508 genes for STKs in the 13 to 14.8 Mb genomes of *Sorangium cellulosum* strains (Han et al. 2013).

As a base for an investigation of the occurrence, abundance, and composition of STKs in the *Planctomycetota* and related bacterial species, a solid phylogeny is needed. Here, we have inferred a concatenated core protein phylogeny to determine the position of “*Ca.* Uabimicrobium amorphum” and *Saltatorellus* species in relation to other members of the *Planctomycetota* phylum and the PVC superphylum for which high quality genomes are available. The obtained phylogeny was used as a framework for a study of the diversification processes in “*Ca.* Uabimicrobium amorphum” in comparison to *Saltatorellus* species and *Planctomycetota*. The evolutionary dynamics of genes in these lineages, with a specific focus on the abundance, domain architecture, and evolution of STKs in members of the PVC superphylum are presented.

## Methods

A dataset of 45 species from the *Planctomycetota* phylum was downloaded from NCBI GenBank (February 2021). The species were selected based on their genome assembly completeness and to include the previously described major clades (“*Ca.* Brocadiales”, *Phycisphaera*, *Isosphaeraceae*, *Pirellula* clade, *Bythopirellula* clade, *Gemmataceae*, *Saltatorellus* clade, *Gimesia* clade) in the *Planctomycetota* phylum (Wiegand et al. 2020). For the *Saltatorellus* clade, we included all of the available genomes and for “*Ca.* Brocadiales” we included high quality genomes obtained from enrichment cultures. We also included the genomes of “*Ca.* Uabimicrobium amorphum”, *Thermogutta terrifontis*, *Planctomycetes bacterium* Pan216, and *Planctomycetes bacterium* 10988, which have not been associated with any of the clades mentioned above. To this dataset, we added outgroup species from the *Verrucomicrobiota* phylum and the *Chlamydiota* phylum for which closed genomes were available at the time of the download, yielding a total of 65 species (supplementary table S1, Supplementary Material online).

### Orthologues Clustering

The proteomes of the 65 selected species (supplementary table S1, Supplementary Material online) were clustered into orthogroups using OrthoFinder2 v2.3.3 (Emms and Kelly 2019) with default parameters, and Diamond v0.9.30.131 (Buchfink et al. 2014) was used for the all-against-all homology search. The proteomes were also clustered with OrthoMCL (Li et al. 2003) using a previously described alignment length filter to reduce the clustering of proteins of different lengths to the same cluster (Mahajan et al. 2020b).

### Species Phylogeny

Proteins encoded by single-copy genes present in at least 95% of the taxa based on the OrthoFinder2 clustering were aligned with MAFFT v7.245 using the L-INS-I option (Katoh and Standley 2013). Three different supermatrix alignment were constructed by (i) no alignment trimming, (ii) trimming the alignments of the individual proteins using BMGE with the Blosum30 model before concatenation, and (iii) trimming the alignment after concatenation using the stationary trimming mode in BMGE (-s FAST -h 0:1 -g 1). This third trimming removes sites from the alignment until the remaining sites are compositionally homogeneous (Criscuolo and Gribaldo 2010). Maximum-likelihood phylogenies were calculated for each of the supermatrix alignments with IQ-Tree v2.0.3 (Minh et al. 2020) using the PMSF approximation (Wang et al. 2018) of the LG + C60 + F + G4 model using 100 nonparametric bootstrap (NPB) replicates.

### Test of Compositional Bias

To test the possible effect of compositional biases on the species phylogeny, four different supermatrix alignments were constructed based on different biases known to affect species phylogenies. First, the standard deviation of the fractions of sites coded by G and C in the first two codon-positions (aminoGC) for each taxon was calculated for each of the single-copy genes using the alignment_pruner.pl script obtained from https://github.com/novigit/davinciCode (commit aea89ef).

Next, the synonymous (dS) and nonsynonymous substitution (dN) frequencies of the single-copy genes were analyzed using codeML in PAML v4.9 (Yang 2007). Protein sequences for the single-copy genes were aligned with MAFFT L-INS-i, and PAL2NAL v14 (Suyama et al. 2006) was used to convert the amino acid alignment to a codon alignment. Four supermatrix alignments were constructed by concatenating the alignment of the 25 proteins encoded by single-copy genes comprising the smallest aminoGC-bias, the lowest dN-values, the largest aminoGC-bias, and highest dN-values, respectively. IQ-Tree was used to calculate phylogenies for each dataset under the LG substitution model with 1,000 ultrafast bootstrap replicates.

### Functional Category Prediction

The proteomes were used as queries in a protein BLAST search against the Clusters of Orthologous Groups (COGs) database (Galperin et al. 2015) using BLAST v2.6.0+ (Altschul et al. 1997, 2005) with the *e*-value cut-off set to 1e−5. A COG category was assigned to a protein if a region in the protein had at least five hits in the COG database with the specified COG category and with no more than 50% of the region classified to another COG category. Nonassigned regions were regions lacking hits (marked by “−”, having multiple COG categories predicted over the same region with 50% or more overlap (marked by “?”), and regions with less than five hits to a COG category in the COG database (marked by “+”).

### Gene Flux Analysis

Count (Csurös 2010) was used to determine the gene flux over the species phylogeny obtained from the maximum likelihood phylogeny of all single-copy protein families using Wagner parsimony together with a gain penalty of 2.

### Identification of Serine/Threonine Protein Kinases

The hmmsearch tool from HMMER 3.3.2 (Eddy 2011) was used to find proteins with the Pfam domain of Pkinase (PF00069) in the PVC proteome dataset, and in the proteomes of *S. cerevisiae*, *S. pombe*, and *C. elegans*. A further refinement of this search was done following the scheme in supplementary fig. S4, Supplementary Material online. In summary, we classified domains as functional if the identified domain contained the catalytic KDD sites (Hanks 2003). If not, we checked if the protein contained fractions of the Pkinase domain and if those fractions together formed a complete Pkinase domain with the KDD sites present. If so, the domain was classified as functional.

### Phylogeny of Serine/Threonine Protein Kinases

The amino acid sequences of functional Pkinase domains from Serine/Threonine Protein kinases in *Planctomycetota* were extracted and aligned using the HMM of the Pkinase domain (PF00069). The alignment was trimmed with BMGE usinge the BLOSUM30 model with entropy cut-off (-h) set to 0.7 and gap threshold (-g) set to 0.8. A phylogeny was calculated with Fasttree v2.1 (Price et al. 2010) under the LG + CAT model.

### Domain and Transmembrane Predictions

InterProScan v5.56-89.0 (Jones et al. 2014) was used to identify associated Pfam-domains in the functional protein kinases. Transmembrane regions were predicted with DeepTMHMM (Hallgren et al. 2022) and an in-house Python script was used to create schematic figures over domain architecture and transmembrane regions.

## Results

### Inference of the Planctomycetota Species Tree

To infer the relative order of divergence of the newly discovered members of the *Planctomycetota* phylum, “*Ca.* Uabimicrobium amorphum” and *Saltatorellus*, we compiled a dataset that includes a selected set of five closed genomes from each of the previously described groups *Phycisphaerae*, *Gemmataceae*, *Isophaeraceae*, *Bythopirellula*, *Pirellula*, and *Gimesia*. To this set, we added genomes from members of *Saltatorellus*, “*Candidatus* Brocadiales” and the single genome of “*Ca.* Uabimicrobium amorphum”, which resulted in a dataset of 45 *Planctomycetota* genomes (supplementary table S1, Supplementary Material online). As outgroups, we selected 20 closed genomes from *Verrumicrobiota* and *Chlamydiota* (supplementary table S1, Supplementary Material online). The proteins of all species were clustered into protein families using OrthoFinder and 114 single-copy orthologs (supplementary table S2, Supplementary Material online) were identified in at least 95% of the taxa. A maximum likelihood phylogeny of the *Planctomycetota* phylum inferred from a concatenated alignment of these proteins provided strong support for the previously recognized groups and indicated a monophyletic relationship between “*Ca*. Uabimicrobium amorphum” and “*Ca*. Brocadiales”, which was also the earliest diverging clade of the *Planctomycetota* phyla (supplementary fig. S1A, Supplementary Material online). A similar topology was obtained when the single protein alignments had been trimmed with BMGE using the Blosum30 model prior to the concatenation (supplementary fig. S1B, Supplementary Material online).

### Removal of Biased Proteins and Sites Favors the Monophyly of “*Ca.* Uabimicrobium Amorphum and *Saltatorellus*

The genomes in the *Saltatorellus* clade have genomic GC-contents of 70%, which is markedly different from those of “*Ca.* Uabimicrobium amorphum” and “*Ca.* Brocadiales”, which have genomic GC-content values of 39% to 43% (supplementary table S1, Supplementary Material online). The genomic GC-content in other *Planctomycetota* species ranges from 50% to 70%. It is known that phylogenies inferred from taxa with a large difference in GC composition tend to cluster taxa with similar GC-content, potentially masking the true phylogenetic signal (Viklund et al. 2012). Rapidly evolving proteins are more vulnerable to GC-bias than slowly evolving proteins, and hence, different topologies might also be anticipated for trees inferred from slowly and rapidly evolving proteins. Also, as the sequence of rapidly evolving genes varies to a greater extent among the species, the risk of misinterpreting phylogenetic signals increases.

**Fig. 1.**
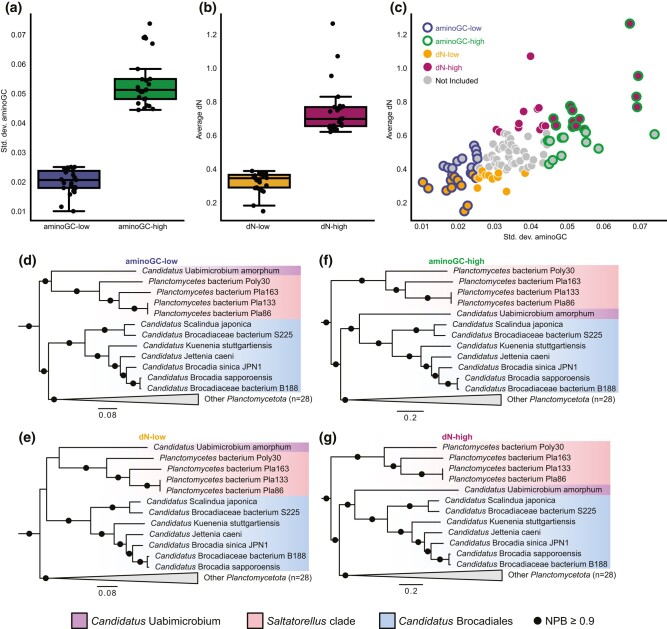
Effect of compositional biases on the *Planctomycetota* phylogeny. a) Distribution of the standard deviation of the fraction of sites coded by GC in the first two codon positions (aminoGC) for each of the 25 single-copy genes included in the aminoGC-low and aminoGC-high datasets. b) Distribution of the average nonsynonymous substitution frequency (dN) for each of the 25 single-copy genes included in the dN-low and dN-high datasets. c) Relationship between aminoGC-bias and dN-value for all single-copy genes. Genes are colored according to the dataset in which they are included. Maximum likelihood phylogenies were inferred from a concatenated alignment of proteins in the (d) aminoGC-low, (e) dN-low, (f) aminoGC-high, and (g) dN-high datasets, respectively. Phylogenies were calculated using IQ-Tree under the LG substitution model with 1,000 ultrafast bootstrap replicates and rooted with species from *Verrucomicrobiota* and *Chlamydiota*. Complete phylogenies that include the outgroup species are shown for all datasets in supplementary fig. S2, Supplementary Material online.

To investigate the effect of AT/GC-bias and substitution rates on the *Planctomycetota* phylogeny, we estimated the nonsynonymous substitution frequencies (dN) for all single-copy orthologous genes and calculated aminoGC values for the proteins encoded by these genes. The single-copy orthologs were then ranked based on mean substitution frequencies and standard deviations of the aminoGC-values (supplementary table S2, Supplementary Material online). From these ranks, four datasets were compiled, one set included the 25 orthologs with the smallest variations in aminoGC, another set contained the 25 orthologs with the largest variations in aminoGC (Fig. 1a), a third set comprised the 25 orthologs with the lowest dN-values, and finally a fourth set containing the 25 orthologs with the highest dN-values (Fig. 1b). The overlap between the datasets was 14 of 25 proteins for both the datasets with small aminoGC-variation and low dN-values and the datasets with large aminoGC-variation and high dN-values (Fig. 1c).

Next, we inferred maximum likelihood phylogenies from concatenated alignments of each of the four datasets. In the phylogeny inferred from the orthologs with the least aminoGC-variation, “*Ca*. Uabimicrobium amorphum” and the *Saltatorellus* taxa formed an early diverging clade with 100% bootstrap support and “*Ca.* Brocadiales”, *Phycisphaerae*, and *Planctomycetia* formed a separate clade with 98% bootstrap support (Fig. 1d; supplementary fig. S2A, Supplementary Material online). This topology was also obtained when the phylogeny was inferred from the proteins with the lowest dN-values, although the bootstrap support value for clustering of “*Ca.* Uabimicrobium amorphum” with *Saltatorellus* was not significant (Fig. 1e; supplementary fig. S2B, Supplementary Material online). In contrast, “*Ca*. Uabimicrobium amorphum” formed a clade with “*Ca.* Brocadiales” with 100% bootstrap support in the phylogeny inferred from orthologs with the largest aminoGC-variation (Fig. 1f; supplementary fig. S2C, Supplementary Material online). Likewise, the tree inferred from proteins with the highest dN-values placed “*Ca.* Uabimicrobium amorphum” in a clade with “*Ca.* Brocadiales” with 100% bootstrap support (Fig. 1g; supplementary fig. S2D, Supplementary Material online).

**Fig. 2.**
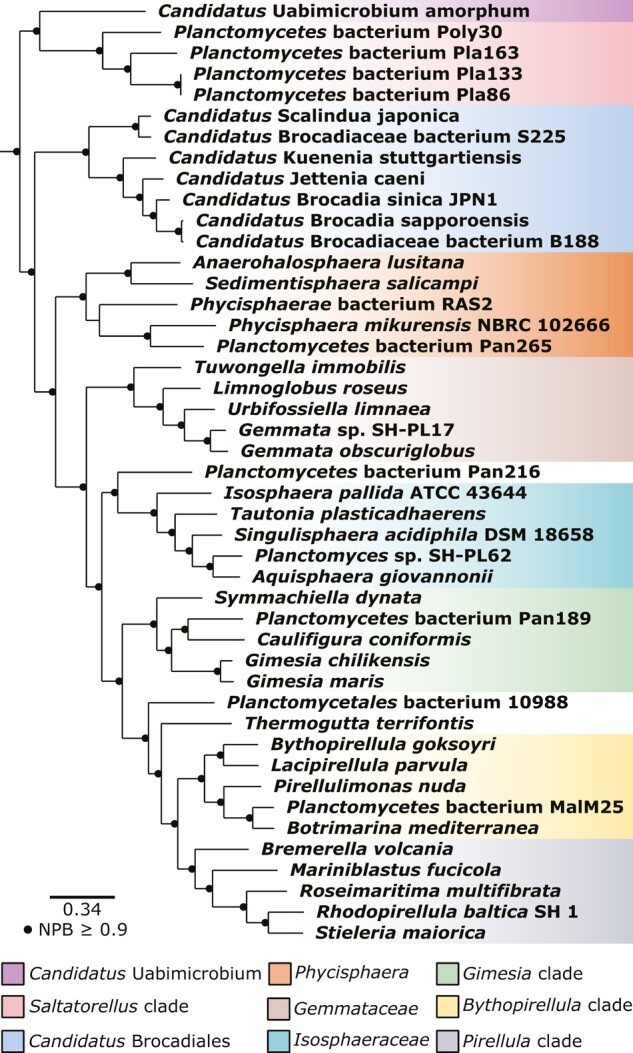
Phylogenetic analysis of the *Planctomycetota* phylum. Maximum likelihood phylogeny of a representative set of taxa in the PVC superphylum inferred from a compositionally homogeneous concatenated alignment of 114 proteins encoded by single-copy genes. The alignment was trimmed using the stationary trimming mode in BMGE, resulting in 44,818 sites. The phylogeny was calculated with IQ-Tree under the posterior mean site frequency (PMSF) approximation of LG + C60 + F + G with 100 NPB replicates and rooted with species from *Verrucomicrobiota* and *Chlamydiota*. The complete phylogeny, including outgroup species is shown in supplementary fig. S1C, Supplementary Material online.

Finally, we inferred a maximum likelihood phylogeny with the posterior mean site frequency (PMSF) approximation of the LG + C60 + F + G substitution model on a site-heterogenous trimmed alignment of all 114 single-copy orthologs. The obtained topology indicated that “*Ca*. Uabimicrobium amorphum” forms a clade with *Saltatorellus* with 100% bootstrap support, while the *Ca.* Brocadiales clade clustered with the remaining *Planctomycetota* species (Fig. 2; supplementary fig. S1C, Supplementary Material online). Thus, the tree topologies obtained from the least aminoGC-biased and the slowest evolving proteins concur with the species tree topology obtained from the concatenated alignment of all 114 single-copy orthologs after removal of compositionally heterogeneous sites. We take these results as strong support for the hypothesis that “*Ca.* Brocadiales” forms a monophyletic clade with the previously described *Planctomycetota* species, while the *Saltatorellus* species and “*Ca.* Uabimicrobium amorphum” represents two earlier-diverging sister lineages. To our knowledge, this is the first study to publish the phylogeny of *Planctomycetota* with both “*Ca*. Uabimicrobium amorphum” and the *Saltatorellus* clade.

### Protein Family Expansion

To identify protein families that might have contributed to the diversification of “*Ca.* Uabimicrobium amorphum” and the *Saltatorellus* clade, we estimated the expansion and loss of protein families in these two lineages. For this analysis, the proteomes of the selected species in the PVC superphylum (supplementary table S1, Supplementary Material online) were clustered into a total of 29,543 protein families using OrthoMCL in combination with an alignment length filter. In “*Ca.* Uabimicrobium amorphum”, 3,988 proteins of the 6,660 annotated proteins were members of 2,844 protein families, of which 234 protein families were unique to this species. In *Saltatorellus*, 1,837 protein families contained proteins from all species in the clade, of which 417 protein families only contained homologs from this clade. The remaining 2,672 proteins in “*Ca.* Uabimicrobium amorphum” and 2,062 proteins in *Saltatorellus* did not have homologs in our dataset and were therefore not included in any protein family, and these proteins are referred to as singletons.

**Fig. 3.**
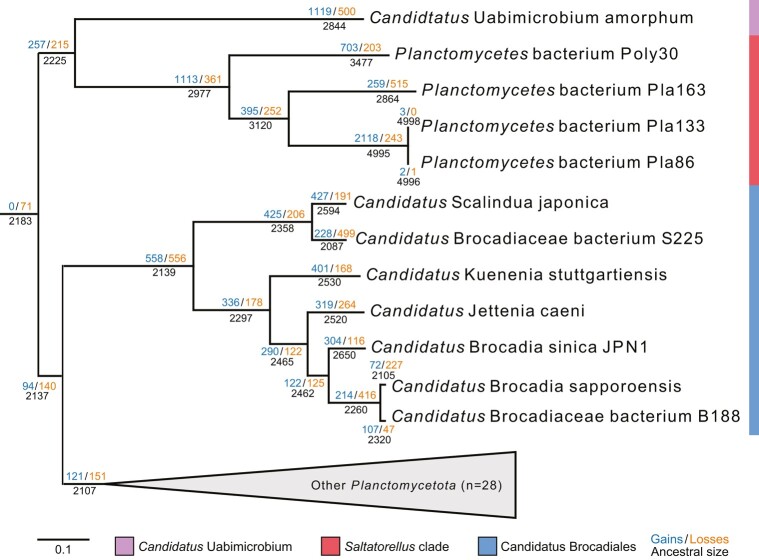
Analysis of the flux of protein families in early-diverging clades of *Planctomycetota*. Labels above the branches indicate the number of gained and lost protein families. Labels below the branches indicate the total number (black) of protein families. The clustering of protein families was done using OrthoMCL with an alignment length filter. The gene flux was computed using a parsimonious method over the maximum likelihood phylogeny presented in supplementary fig. S1C, Supplementary Material online. The gains and losses at all branches in the tree are shown in supplementary fig. S3, Supplementary Material online.

The results of the gene flux analysis (Fig. 3; supplementary fig. S3, Supplementary Material online), which was done using a parsimony method in Count, showed a predicted gain of 257 protein families and a loss of 215 protein families on the node to the clade comprising “*Ca.* Uabimicrobium amorphum” and *Saltatorellus*. Interestingly, the analyses indicated the gain of 1,119 and 1,113 novel protein families in “*Ca.* Uabimicrobium amorphum” and in the ancestor the *Saltatorellus* clade, respectively, whereas the losses were estimated to only 500 protein families in “*Ca.* Uabimicrobium amorphum” and 361 protein families in the common ancestor of the *Saltatorellus* clade. Thus, both lineages featured a net gain of protein families. In contrast, the gain of 558 novel protein families in the common ancestor to “*Ca.* Brocadiales” was balanced by the loss of 556 protein families, and thus no net gain of protein families was inferred in the common ancestor of this clade.

### Functional Categorization of Expanded Protein Families

**Fig. 4.**
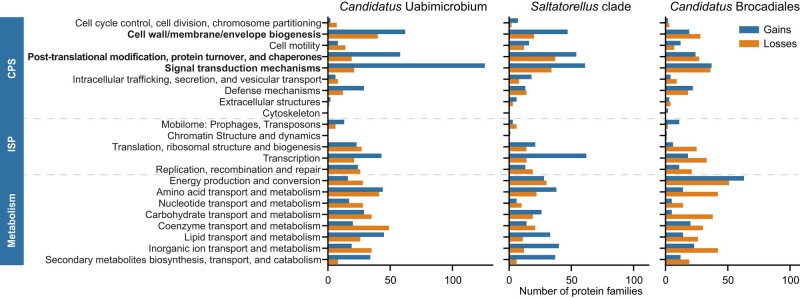
Functional categorization of gained and lost protein families. The distribution of functional categories (COGs) is shown for the gained and lost protein families in “*Ca.* Uabimicrobium amorphum”, the *Saltatorellus* clade, and “*Ca.* Brocadiales”. The COGs are grouped into cellular processes and signaling (CPS), information storage and processing (ISP), and metabolism. Examples of COGs with a higher number of gained than lost protein families in “*Ca.* Uabimicrobium amorphum” are marked in bold.

To learn more about the putative functions of the variably present protein families, we assigned the gained and lost protein families to functional categories (Fig. 4). The results indicated a large gain of 124 protein families related to the signal transduction category in “*Ca.* Uabimicrobium amorphum” (supplementary table S3, Supplementary Material online) as compared to the loss of only 21 such protein families, summing up to a total gain of more than 100 protein families in this category. For the *Saltatorellus* clade, there was also a large, albeit less biased, turnover of protein families related to signal transduction functions, including the gain of 61 (supplementary table S4, Supplementary Material online) and the loss of 34 such protein families, respectively. There was also a relatively higher gain of proteins in the functional categories “Cell wall/membrane/envelope biogenesis” and “Post-translational modification, protein turnover, and chaperones” among the gained families in both *Saltatorellus* and “*Ca.* Uabimicrobium amorphum”. The ancestor of the “*Ca.* Brocadiales” clade showed a remarkably different gain-loss profile, with for example a large turnover of proteins related to the “Energy production and conversion”, which featured 63 protein family gains and 51 protein family losses. In all other categories related to metabolism, the losses outweighed the gains. Specifically, the category “Amino acid transport and metabolism” and “Carbohydrate transport and metabolism” featured losses of 42 and 38 protein families compared to 14 and 5 gains, respectively. In all other functional categories, there was no outstanding bias or overall loss of protein families.

The largest number of protein families in the “*Ca*. Uabimicrobium amorphum” proteome belong to the signal transduction category, of which most are serine/threonine kinases with the PF00069 domain. Consistently, a large fraction, 66 of the 124 protein families in the gained signal transduction category were serine/threonine protein kinases with the PF00069 domain (supplementary table S3, Supplementary Material online). Likewise, 19 of the 61 gained protein families in the signal transduction category *Saltatorellus* were serine/threonine kinase (supplementary table S4, Supplementary Material online). We found this very interesting, as we hypothesized that these gained kinases might provide a clue into the unique behaviors of these taxa. The large numbers alone strongly indicate that these functions are important for these bacteria.

### Distribution/Abundance and Flux of STKs in the PVC Superphylum

**Fig. 5.**
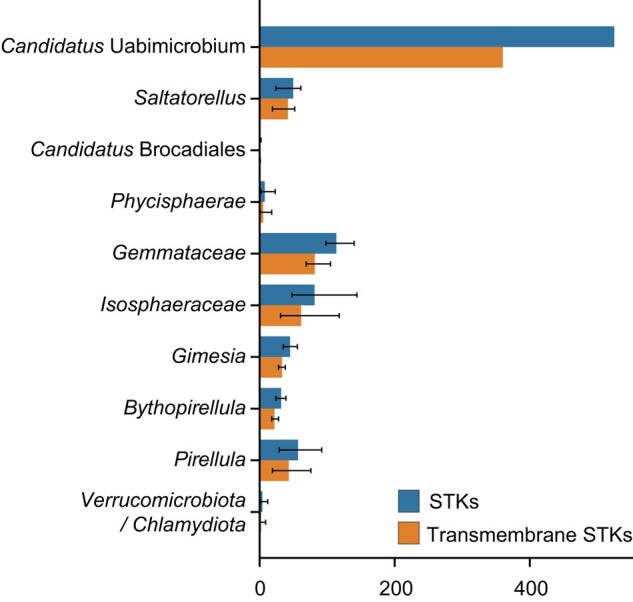
Distribution of Ser/Thr protein kinases in the *Planctomycetota*. The average total number of STKs of which a subset are transmembrane proteins for the different clades in the *Planctomycetota* phylum. Error bars represent the highest and lowest total number of STKs and transmembrane STKs in the different clades.

As “*Ca.* Uabimicrobium amorphum” and *Saltatorellus* both contain several singletons and as not to limit the results to proteins clustered into families, we broadened our search and identified the Ser/Thr kinase proteins with an HMM domain profile search for the catalytical Pkinase domain (Pkinase domain, PF00069) in our representative proteome dataset of species in the PVC superphylum (supplementary fig. S4, Supplementary Material online). We further investigated the functionality of these predicted proteins by checking for the canonical K, D1, and D2 (KDD) sites in their Pkinase domains (supplementary table S5, Supplementary Material online). In total, we identified as many as 610 putative STKs in the proteome of “*Ca.* Uabimicrobium amorphum”, of which 525 most likely correspond to functional kinases, as the KDD sites can be found in their catalytical Pkinase domain (Fig. 5; supplementary table S6, Supplementary Material online). Much lower numbers were predicted in the other groups, with only 98 to 140 STKs in the proteomes of the *Gemmataceae* and about 20 to 60 such proteins in the proteomes of *Pirellula*, *Bythopirellula*, and *Saltatorellus* (Fig. 5; supplementary table S6, Supplementary Material online) No or very few STKs were predicted in the proteomes of “*Ca.* Brocadiales”, *Phycisphaera,* and the outgroup species. After normalization for proteome size, the relative fractions of STKs ranged from 0.02% to 8% in these bacterial groups, with “*Ca*. Uabimicrobium amorphum” as a clear outlier.

**Fig. 6.**
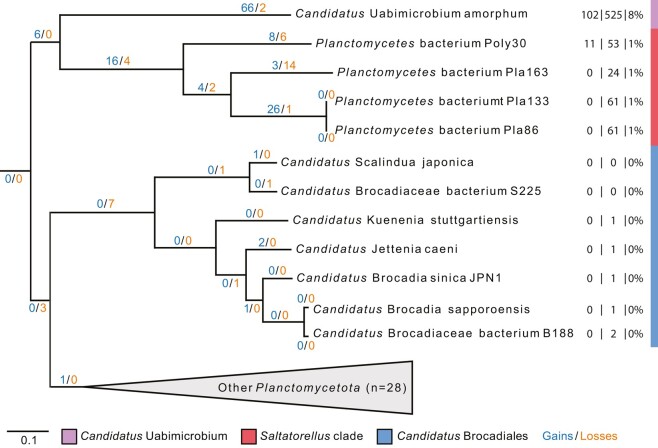
Gene flux of Ser/Thr protein kinase families in early diverging clades of the *planctomycetota*. The number of gained and lost protein families that contain STKs in “*Ca.* Uabimicrobium amorphum”, the *Saltatorellus* clade, and “*Ca.* Brocadiales”. The gene flux was computed using a parsimonious method over the maximum likelihood phylogeny presented in supplementary fig. S1C, Supplementary Material online. Numbers to the right of the taxa indicate the number of unique STKs, the total number of STKs, and the fraction of STKs in the proteome, respectively. Gains and losses of STKs over the complete phylogeny are shown in supplementary fig. S5, Supplementary Material online.

The large variation in STK abundance was also confirmed by an analysis of the flux of protein families that contain the predicted STKs across the species tree (Fig. 6; supplementary fig. S5, Supplementary Material online). In “*Ca*. Uabimicrobium amorphum”, a gain of 66 and loss of 2 protein families for STKs was inferred as compared to 16 gained and 4 lost STK families on the ancestral node to *Saltatorellus* and 28 gains and no losses on the ancestral node to the *Gemmataceae*. These gains were followed by much more recent gains and losses of STKs within both of these two clades. The high number of gained STKs in some of the *Planctomycetota* groups contrasts with no or few, and almost constant, numbers of STKs in “*Ca.* Brocadiales” and the outgroup species.

### Phyletic Distribution Pattern of Accessory Domains in the STKs

The large majority of the predicted STKs, typically 70% to 80%, contained transmembrane regions, irrespective of whether the proteomes contained only a few or several hundred STKs (Fig. 5; supplementary table S6, Supplementary Material online). Most of these contained a single transmembrane region, but some STKs were predicted to contain as many as 18 transmembrane regions. This suggests that most of the identified STKs are membrane-spanning proteins.

**Fig. 7.**
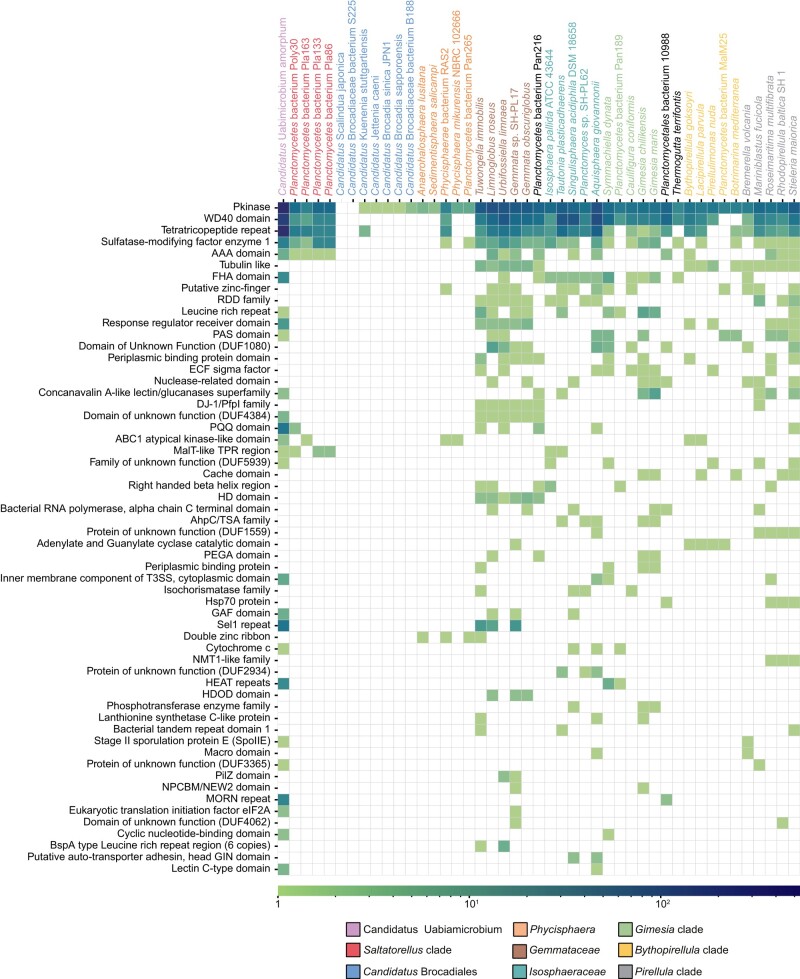
Phyletic distribution pattern of accessory domains in the Ser/Thr protein kinases in the *Planctomycetota*. Heatmap showing the phyletic distribution pattern and the relative abundance of the accessory Pfam-domains that are present in two or more species in the *Planctomycetota*. Species have been sorted based on the inferred phylogeny of the *Planctomycetota* shown in Fig. 2.

The occurrence and abundance of Pfam domains associated with the Pkinase domain were investigated for both the membrane and the cytoplasmic STKs. On average, about half of the STKs in the PVC superphylum were found to contain associated domains. In total, we identified 132 Pfam domains in these proteins, which were sorted into 105 functional domain groups (supplementary table S7, Supplementary Material online). A heatmap displaying the occurrence and abundance of these accessory domain groups across the PVC phylogeny revealed a scattered phyletic distribution pattern for about half of the 105 domain groups which were identified in more than one of these species (Fig. 7; supplementary table S7, Supplementary Material online). Among these, the WD40 and the tetratricopeptide (TPR) repeat domains were outstanding with regard to both occurrence and abundance. The other half of the 105 accessory domains were only identified in a single protein in a single species, and 19 of these domains were uniquely associated with STKs in “*Ca.* Uabimicrobium amorphum” (supplementary table S7, Supplementary Material online). Thus, the genome of “*Ca.* Uabimicrobium amorphum” contained not only the largest number and the highest relative fraction of STKs but also the most unique and the most diverse set of STKs in the PVC phylum.

### Domain Architectures of the STKs

**Fig. 8.**
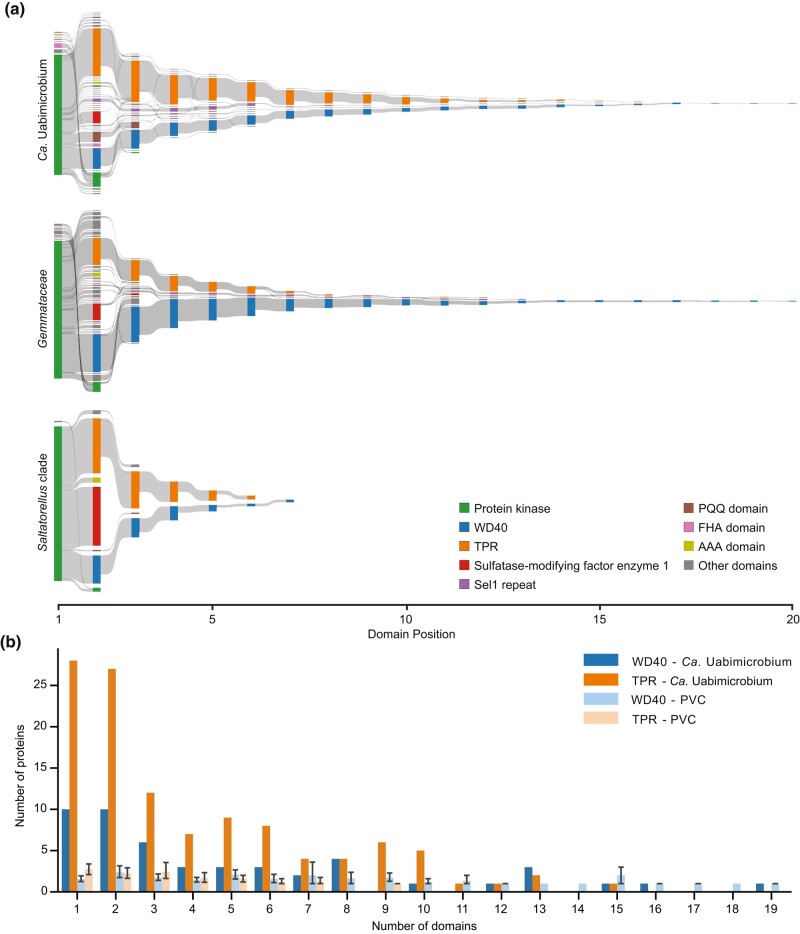
Domain architectures of multidomain Ser/Thr protein kinases. a) Overview of domain architectures for multidomain STKs in “*Ca.* Uabimicrobium amorphum”, *Gemmataceae*, and the *Saltatorellus* clade. b) The number of TPR and WD40 domains per protein is plotted against the number of STKs that contain them in “*Ca*. Uabimicrobium amorphum” in comparison to other species in the PVC superphylum.

The complete set of 105 accessory domains was combined with the Pkinase domain and transmembrane regions to form a total of 307 different protein architectures in the identified STKs in the *Planctomycetota* and the outgroup species affiliated with *Verrucomicrobiota* and *Chlamydiota*. (supplementary fig. S6 and supplementary table S8, Supplementary Material online). From the 525 putatively functional STKs in “*Ca.* Uabimicrobium amorphum”, 242 proteins contained only the Pkinase domain. About 60% of the remaining 283 STKs contained either the WD40 or the TPR domain at their C-terminal ends (Fig. 8a). Similar domain architectures were also observed for the STKs in members of *Gemmataceae* and *Saltatorellus* (Fig. 8a). In total, the STKs of “*Ca.* Uabimicrobium amorphum” contained 467 TPR domains in 115 proteins and 250 WD40 domains in 49 proteins (i.e. four to five domains per protein on average). The multiple copies of the TPR and WD40 domains formed long tandem arrays of up to 15 and 19 domains per protein, respectively (Fig. 8b; supplementary table S8, Supplementary Material online). Thus, although “*Ca.* Uabimicrobium amorphum” contained twice as many STKs with TPR domains than WD40 domains, the WD40 domains formed longer arrays.

The prediction of transmembrane helices and signal peptides further allowed us to determine the intracellular location of the Pkinase domains as well as of the associated domains in the transmembrane-spanning STKs. The Pkinase domains were in all proteins predicted to be located within the cytoplasmic space (supplementary fig. S7, Supplementary Material online), which is concordant with the fact that the periplasmic space is devoid of ATP, the substrate used by this domain. The most commonly associated domains, the TPR and the WD40 repeat domains, were mostly located in the periplasmic space and also mostly present in single-spanning transmembrane proteins. The third most abundant domain, sulfatase modifying enzyme 1 domain, which is present in proteins that activate sulfatases, was present in almost all species and mostly located on the periplasmic side in single-spanning transmembrane STKs. The domain DUF1080 was the most abundant domain among those exclusively present on the periplasmic side of single-spanning transmembrane STKs, whereas the tubulin-like domain was the most common associated domain among those solely present in cytoplasmic STKs. However, none of the latter two domains were present in “*Ca*. Uabimicrobium amorphum” or *Saltatorellus.*

In an attempt to infer the events that have generated such a diversity of proteins, we performed a phylogenetic analysis of the STKs in the *Planctomycetota* based on the Pkinase domain alone. Although the tree contained a few thousand sequences and as such was not well resolved, the topology provided indications for several lineage-specific expansion events (supplementary fig. S8, Supplementary Material online). For example, a few clades contained more than 50 Pkinase domains from “*Ca*. Uabicrobium amorphum”, suggesting that the expansions in this species have largely occurred by duplications and/or transfers within the lineage. There were however no indications that STKs with similar domain arrangement in different species formed highly supported clades, suggesting that similar domain architectures may have originated multiple times independently.

For comparison, the *Saccharomyces cerevisiae* S288C and *Schizosaccharomyces pombe* genomes contained 112 and 102 genes for STYKs, respectively, while *Caenorhabditis elegans* harbored 617 genes for STYKs, which correspond to about 2% of their proteomes (supplementary table S6, Supplementary Material online). In contrast to the high fraction of membrane-spanning STKs in the *Planctomycetota*, only 1% to 11% of the STYKs in these eukaryotic species were predicted to contain transmembrane regions (supplementary table S6, Supplementary Material online), suggesting that the majority are located in the cytoplasm. In total, 69 different associated domains were identified, which altogether formed 98 different domain architectures (supplementary fig. S9 and supplementary table S9, Supplementary Material online). About 7% of the STYKs in *C. elegans* contained long N-terminal arrays with up to 30 Ig-domains. Except for the ankyrin repeat domain, the cyclic nucleotide-binding domain and the haspin-like domain, the associated domains in the STYKs of yeast and *C. elegans* differed from those in the STKs of the *Planctomycetota*.

## Discussion

In this study, we analyzed the phylogenetic placement of two bacterial lineages with cell shape-shifting and phagocytotic abilities, *Saltatorellus* and “*Ca.* Uabimicrobium amorphum”, respectively. We found that these two groups formed a monophyletic clade that diverged prior to “*Ca*. Brocadiales”, *Phycisphaerae*, and *Planctomycetia* in the PVC superphylum. A gene flux analysis revealed a massive gain of signal transduction systems in the “*Ca*. Uabimicrobium amorphum” lineage, and it was estimated that 8% of its proteome consists of STKs, which represents the highest known fraction of genes for STKs in a bacterial genome. By comparing the contents and architectures of STKs in bacteria and STYKs in eukaryotes, we can start deciphering whether, and if so how, the evolutionary dynamics of these kinases are linked to features that are normally ascribed to eukaryotes, such as complex intracellular structures, multicellularity, and/or phagocytotic abilities.

As a basis for the study, we inferred the first solid and highly supported phylogeny of taxa in the PVC phylum that includes both “*Ca.* Uabimicrobium amorphum” and *Saltatorellus*. Several attempts have been made previously to place *Saltatorellus* and “*Ca*. Uabimicrobium amorphum” in relation to members of the *Planctomycetota*, but the results have been inconclusive. Analyses based on 16S rRNA sequence data indicated that “*Ca.* Uabimicrobium amorphum” clusters with “*Ca.* Brocadiales” (Shiratori et al. 2019; Lodha et al. 2021). The monophyly of this clade was also supported by a tree based on concatenated proteins, but *Saltatorellus* was not included in the analyses (Shiratori et al. 2019; Lodha et al. 2021). Another study suggested that the *Saltatorellus* clade branched outside “*Ca*. Brocadiales”, but the tree did not include “*Ca.* Uabimicrobium amorphum” (Wiegand et al. 2019). A third 16S rRNA analysis indicated that “*Ca.* Brocadiales” diverged prior to “*Ca.* Uabimicrobium amorphum” and *Saltatorellus,* but without significantly high bootstrap support (Mahajan et al. 2020a).

Phylogenies over the *Planctomycetota* phylum have also been inferred based on marker genes and the inclusion of a large diversity of metagenome-assembled genomes (Hägglund et al. 2023). In those phylogenies, the position of “*Ca*. Uabimicrobium amorphum” depended on how the dataset was constructed. One of the phylogenies positioned “*Ca*. Uabimicrobium amorphum” in the same clade as *Saltatorellus,* while another phylogeny placed them in the same clade as “*Ca.* Brocadiales”. However, none of the clades were well supported by ultrafast bootstrap. It is also worth pointing out that the alignments underlying the phylogenies were not controlled for assembly errors or compositional biases. Nevertheless, these trees suggest that there is a large diversity of species from early-diverging members of the *Planctomycetota* phylum that have not yet been cultivated.

The phylogenies presented in this paper were based on orthologous proteins from cultured species from the *Planctomycetota,* for which high-quality, complete genomes were available to allow quantitative estimates of STKs and detailed analyses of their domain composition patterns. With a genomic GC-content of only about 40%, “*Ca.* Brocadiales” and “*Ca.* Uabimicrobium amorphum” differ markedly from *Saltatorellus* and other *Planctomycetota,* which have genomic GC-contents in the range of 50% to 70%. Hence, the clustering of “*Ca.* Uabimicrobium amorphum” with “*Ca*. Brocadiales”, as observed previously (Shiratori et al. 2019; Lodha et al. 2021) is most likely an effect of their relatively higher AT-content. Indeed, we only observed clustering of “*Ca.* Uabimicrobium amorphum” with “*Ca*. Brocadiales” in phylogenetic inferences based on proteins that are highly affected by taxa-specific mutation biases, whereas “*Ca.* Uabimicrobium amorphum” clustered with *Saltatorellus* to the exclusion of “*Ca*. Brocadiales” in trees inferred from highly conserved proteins with low levels of variation in GC-content among the taxa included in the analysis. This strongly suggests that *Saltatorellus* and “*Ca*. Uabimicrobium amorphum” form a monophyletic clade, sister to the remaining *Planctomycetota.*

Based on this phylogeny, we re-examined the flux of genes in the *Planctomycetota* (Mahajan et al. 2020b), but now also including “*Ca*. Uabimicrobium amorphum” and *Saltatorellus*. The branch leading to the class *Planctomycetia* featured a net gain of about 1,000 novel protein families, similar to the number of protein families gained on the branches to the ancestors of “*Ca*. Uabimicrobium amorphum” and *Saltatorellus*, respectively. Functional categorization of the gained families in “*Ca*. Uabimicrobium amorphum” revealed a much higher relative gain of signal transduction proteins in this species than in any of the other lineages, of which the majority comprised protein kinases with the Pkinase domain. With the aid of a hidden Markov model for STKs, we identified as many as 525 STKs in the proteome of “*Ca*. Uabimicrobium amorphum” as compared to circa 100 to 150 such proteins in the *Gemmataceae* proteomes and 20 to 50 such proteins per proteome in most other clades.

Of the 525 STKs in “*Ca*. Uabimicrobium amorphum”, 54% contain associated protein domains. We propose that the high abundance of STKs with associated domains in this species is mostly due to expansions of already existing domain combinations rather than to functional innovations that are unique to this lineage. This conclusion is based on four observations: Firstly, about 80% of the identified STKs are membrane proteins in all *Planctomycetota* species. Secondly, most of the associated domains show a scattered phyletic distribution pattern and less than 5% of the STKs in “*Ca*. Uabimicrobium amorphum” contain domains that are unique to this lineage. Thirdly, a large fraction of the STKs in “*Ca*. Uabimicrobium amorphum” and the other *Planctomycetota* species contains tandem arrays of either WD40 or TPR domains at the C-terminal ends. This suggests that the composition and architectures of protein domains in the STKs of “*Ca.* Uabimicrobium amorphum” are broadly present in the phylum. Fourthly, our phylogenetic study based on sequence alignments of the pKinase domain revealed several highly supported clades that contained large numbers of STKs from “*Ca*. Uabimicrobium amorphum”, indicative of duplication and/or recombination events within the lineage. The STKs in the species-specific clades contained some of the most abundant associated domains, such as the TPR, WD40 or FHA domains. It has previously been shown that phylogenies of the pKinase domain from other bacterial groups also contained several species-specific clades, suggesting that duplication and/or recombination events within the species represent a common mechanism to expand the number of STKs over short evolutionary time scales (Pérez et al. 2008).

The association of the pKinase domain with the TPR and WD40 domains has also been observed in *Myxobacteria* and a few other bacterial species with more than 15 STKs (Pérez et al. 2008). For example, 26% of the 317 STKs in *S. cellulosum* contain TPR domains at their C-terminal ends (Han et al. 2013), which is comparable to 21% in “*Ca.* Uabimicrobium amorphum”. The function of the TPR domain in these proteins is mostly unknown, but the cytoplasmic PknG protein with TPR domains in *Mycobacterium tuberculosis* (Scherr et al. 2007) and *Corynebacterium glutamicum* (Lisa et al. 2021) have been shown to receive a signal via the membrane protein GlnX from the extracellular GlnH protein, which senses the availability of amino acids. Furthermore, the amino acid residues in the WD40 domains that interact with other molecules in cyanobacterial STKs have been shown to be highly diverged, indicating a diverse set of signal inputs (Hu et al. 2017).

The eukaryotic STYKs contain other types of associated domains and the domain architectures are characteristically different from those of bacteria. For example, the co-occurrence of the Pkinase domain and the WD40 domain was only rarely observed (van Nocker and Ludwig 2003), although WD40 domain proteins represent the most highly abundant protein family in eukaryotes (Mahajan et al. 2020b). It is also notable that the fraction of membrane-spanning STYKs is much lower in the eukaryotes. Taken together, this suggests that the STKs in the *Planctomycetota* and *Myxobacteria* were not acquired by horizontal gene transfer from the eukaryotes, nor were they retained from a shared common ancestor, although the pKinase domain itself may have been ancestrally present. Rather, the dramatic expansions of STKs in eukaryotes and a few bacterial species is likely to reflect convergence.

This raises the question of why the STKs have expanded in only a few bacterial species? It has been shown previously that the number of proteins with the Pkinase domain increases exponentially with genome and proteome sizes (Pérez et al. 2008; Mahajan et al. 2020b). However, genome or proteome size alone is unlikely to explain the lineage-specific expansions in “*Ca.* Uabimicrobium amorphum”, which has five times as many such genes as members of the *Gemmataceae* despite similar genome sizes in the 7 to 10 Mb range. Another hypothesis is that the expansion of STKs in the *Planctomycetota* is related to their complex cellular structures (Arcas et al. 2013). The near absence of genes for STKs in “*Ca.* Brocadiales”, which has a bacterial-like cell envelope with a peptidoglycan cell wall and no invaginations of the cytoplasmic membrane (although it has an organelle), is consistent with this idea. However, also this hypothesis does not explain the extreme expansion of STKs in “*Ca.* Uabimicrobium amorphum”.

In animals, multicellularity has been suggested as a driving force behind the expansion of signal transduction systems (Ocaña-Pallarès et al. 2022). Likewise, the expanded repertoire of STKs in *Myxobacteria* has been suggested to play a role in the coordination of population behaviors related to their multicellular lifestyles (Pérez et al. 2008). We do not know if “*Ca.* Uabimicrobium amorphum” or other members of the *Planctomycetota* may under some conditions form multicellular groups. Notably, *Myxobacteria* are also recognized for their ability to prey on bacteria and fungi (Muñoz-Dorado et al. 2016), which may likewise require the ability to monitor changes in the environment and control cell behaviors accordingly. Most bacterial predators are solitary predators, like *Streptomyces* which is an epibiotic predator that attaches to the prey cells and secrets lytic enzymes to kill its victim (Kumbar et al. 2014; Yamada et al. 2023), or *Bdellovibrio* which divides in the periplasm of its prey from where it secretes hydrolytic enzymes. *Myxobacteria* on the other hand are social predators that exhibit a wolf-pack mode of predation, meaning that these bacteria attack their prey as a group. The hunting strategies and social lives of the *Planctomycetota* remain largely unexplored, but is tempting to speculate that the expansion of STKs in the “*Ca.* Uabimicrobium amorphum” is linked to the need for a coordinated population behavior as in the *Myxobacteria*, perhaps related to a phagocyotic or multicellular lifestyle. Social behaviors, like the ability to form multicellular aggregates, recognize prey cells, ingest bacterial cells and import large macromolecules into the enlarged periplasmic space is likely to select for multiple signal transduction pathways organized into complex network structures.

To summarize, the findings presented in this paper have revealed massive duplications of STKs in “*Ca.* Uabimicrobium amorphum”. Although the atypically high abundance of STKs in this species is a characteristic feature of eukaryotes rather than of bacteria, the high fraction of membrane-spanning STKs and the association with TPR and WD40 domains are typical of bacteria rather than of eukaryotes. This suggests that the massive expansions of STYKs in the eukaryotes and of STKs in a few bacterial species reflect convergent evolution. A testable hypothesis is that the expansion of STKs in “*Ca.* Uabimicrobium amorphum” has been driven by their predation strategies or other social behaviors. Future studies should aim at exploring the social lifestyles and hunting strategies of this unique group of bacteria, so as to place the role of the expanded repertoire of STKs in an ecological context.

## Supplementary Material

evae068_Supplementary_Data

## Data Availability

The raw data of alignments and phylogenies are available in supplementary data, Supplementary Material online file 1, Supplementary Material online. Additional datasets and the underlying code for this project are available at the SciLifeLab Data Repository under 10.17044/scilifelab.c.6390417.
