# Supplementary material for: Phylogeny and Expansion of Serine/Threonine Kinases in Phagocytotic Bacteria in the Phylum Planctomycetota
Source: Genome Biol Evol. 2024 Mar 28;16(4):evae068. doi: 10.1093/gbe/evae068 (PMC11032199; doi:10.1093/gbe/evae068)

SUPPLEMENTARY FIGURE AND TABLE LEGENDS

Phylogeny and Expansion of Serine/Threonine Kinases in Phagocytotic Bacteria in the Phylum *Planctomycetota*

Anna Odelgard[1*], Emil Hägglund[1*], Lionel Guy[2], Siv G. E. Andersson[1]

SUPPLEMENTARY FIGURES

Fig. S1. Maximum likelihood phylogenies of a representative set of taxa in the PVC superphylum inferred from a concatenated alignment of 114 proteins encoded by single-copy genes. The alignment was (a) not trimmed, resulting in 60,461 amino acid sites, (b) trimmed for each protein separately prior to the concatenation with the default mode of BMGE using the Blosum30 model, resulting in 29,799 amino acid sites and (c) trimmed after the concatenation with the stationary trimming mode in BMGE, resulting in 44,818 amino acid sites. The phylogeny was calculated with IQ-Tree under the posterior mean site frequency approximation of LG+C60+F+G with 100 non-parametric bootstrap (NPB) replicates and rooted with species from *Verrucomicrobiota* and *Chlamydiota*.

Fig. S2. Maximum likelihood phylogenies of *Planctomycetota* species based on concatenated alignments of different sets of proteins encoded by single-copy genes. The phylogenies were inferred from 25 proteins with (a) low aminoGC-bias, (b) low nonsynonymous substitution frequencies, (c) high aminoGC-bias and (d) high nonsynonymous substitution frequencies. The trees were calculated with IQ-Tree under the LG substitution model with 1000 ultrafast bootstraps. All taxa, including outgroup species affiliated with *Verrucomicrobiota* and *Chlamydiota,* are included in the tree presentations.

Fig. S3. Analyses of the flux of protein families in the PVC superphylum. Labels on the branches indicate the number of gains (green) and losses (red). The clustering of protein families was done using OrthoMCL with the described alignment length filter. The gene flux was computed using a parsimonious method over the maximum likelihood phylogeny presented in supplementary fig. S1c.

Fig. S4. Schematic illustration of the workflow to identify Serine/Threonine kinases with the Pkinase domains and the accessory domains.

Fig. S5. Analyses of the flux of Serine/Threonine kinase (STK) protein families in the PVC superphylum. Numbers on top of the branches indicate the number of gains (green) and losses (red). Numbers below the branches refer to singletons, the total number of proteins and the total number of lost proteins. The flux of STK protein families was computed using a parsimonious method over the maximum likelihood phylogeny presented in supplementary fig. S1c.

Fig. S6. Schematic overview of the 307 protein architectures observed in STKs that contain the protein kinase domain (PF00069) in the *Planctomycetota* and the outgroup species affiliated with *Verrucomicrobiota* and *Chlamydiota.* Each row represents a protein architecture with the number of proteins having this domain architecture displayed on the right-hand side. For visualization purposes, the lengths of the domains and sequences are not shown to scale. The most common domains are marked by different colours, and coloured circles represent the phylogenetic clades in which the protein architecture has been identified. Transmembrane and signal peptide regions are marked by smaller grey boxes. The direction of the transmembrane regions is marked with c_TM_n and n_TM_c, where c represents the cytoplasmic space and n represents the periplasmic space. Domains separated by more than 100 amino acids are separated by a longer black line.

Fig. S7. Cellular localization of the Pkinase-domain (PF00069) and their associated domains in the STKs in the *Planctomycetota* based on predictions of transmembrane segments and signal peptides with DeepTMHMM. Bars to the left indicate a cytoplasmic localization of the domain, and bars to the right indicate a periplasmic localization of the domain. The domains have been color-coded based on whether they reside in STKs with no transmembrane segment (blue), a single transmembrane segment (orange), or more than one transmembrane segment (green).

Fig. S8. Phylogenetic analysis of Serine/Threonine kinases in the PVC-superphylum. Maximum likelihood phylogeny based on multiple sequence alignment of the identified Pkinase domains in the STKs in the *Planctomycetota* and the outgroup species affiliated with *Verrucomicrobiota* and *Chlamydiota.* The taxa have been coloured as in supplementary figures S1 and S2. The names of the taxa include the species name, the domains contained in the protein, and the number of the domain architecture displayed in supplementary figure S6. The phylogeny was calculated with Fasttree under the LG+CAT substitution model. Support values were estimated with the Shimodaira-Hasegawa test as implemented in Fasttree.

Fig. S9. Schematic overview of the 98 identified domain architectures observed in STYKs that contain the protein kinase domain (PF00069) in *S. cerevisiae. S. pombe* and *C. elegans*. Each row represents a protein architecture with the number of proteins having this domain architecture displayed on the right-hand side. For visualization purposes, the lengths of the domains and sequences are not shown to scale. The ten most common domains are marked by different colours, and coloured circles represent the species in which the protein architecture has been identified. Transmembrane and signal peptide regions are marked by smaller grey boxes. The direction of the transmembrane regions is marked with c_TM_n and n_TM_c, where c represents the cytoplasmic space and n represents the outside of the cell. Domains separated by more than 100 amino acids are separated by a longer black line.

SUPPLEMENTARY TABLES

Table S1. Dataset used for the phylogenies, including species names, genome sizes and accession numbers.

Table S2. Single-copy genes used for the phylogenetic inferences. Gene annotations, accession numbers, aminoGC, nonsynonymous substitution frequencies and phylogenetic datasets in which the genes were included.

Table S3. Gained proteins classified into the COG functional category signal transduction (T) in "*Ca.* Uabimicrobium amorphum". The table includes Pfam-domain annotations.

Table S4. Gained proteins classified into the COG functional category signal transduction (T) in *Saltatorellus.* The table includes Pfam-domain annotations.

Table S5. Summary of Pkinase-domain (PF000069) containing proteins with KDD-sites identified in the *Planctomycetota*, the outgroup species affiliated with *Verrucomicrobiota* and *Chlamydiota,* and in *S. cerevisiae* S288C, *S. pombe* and *C. elegans*.

Table S6. The number of proteins that contain the Pkinase domain and the number of such proteins that also contain transmembrane regions in the *Planctomycetota*, the outgroup species affiliated with *Verrucomicrobiota* and *Chlamydiota,* and in *S. cerevisiae* S288C, *S. pombe* and *C. elegans*.

Table S7. The type and total number of domains associated with the Pkinase domain in the STKs identified in the *Planctomycetota* and the outgroup species affiliated with *Verrucomicrobiota* and *Chlamydiota*.

Table S8. Total number of proteins and domain annotations for the 307 different domain architectures identified in the STKs in the *Planctomycetota* and the outgroup species affiliated with *Verrucomicrobiota* and *Chlamydiota*. The table includes information about the taxonomic groups in which a given domain architecture was identified.

Table S9. Total number of proteins and domain annotations for the 98 different domain architecture identified in the STYKs in *S. cerevisiae* S288C, *S. pombe* and *C. elegans*. The table includes information about the taxonomic groups in which a given domain architecture was identified.

Supplementary Fig. S1a

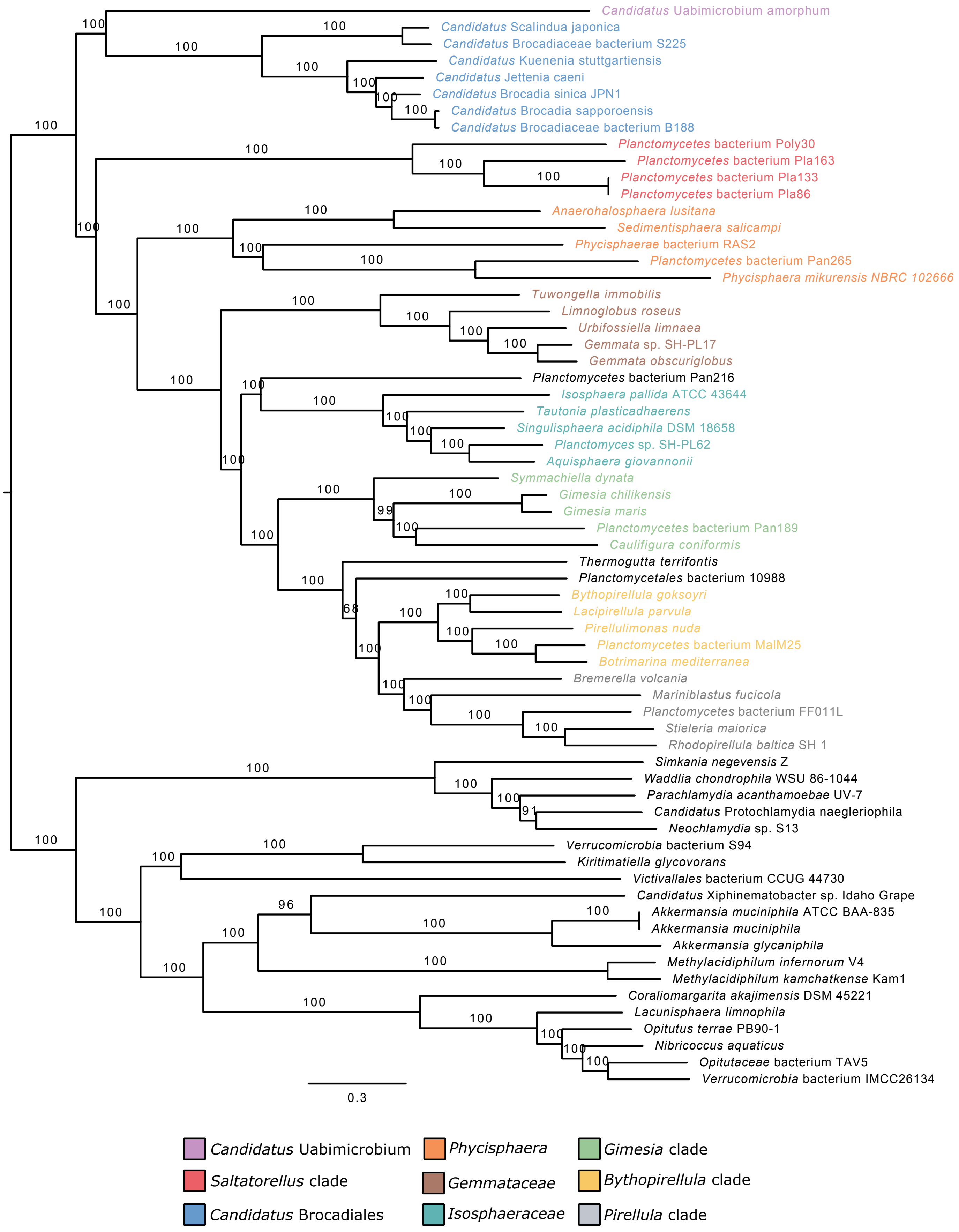

Supplementary Fig. S1b

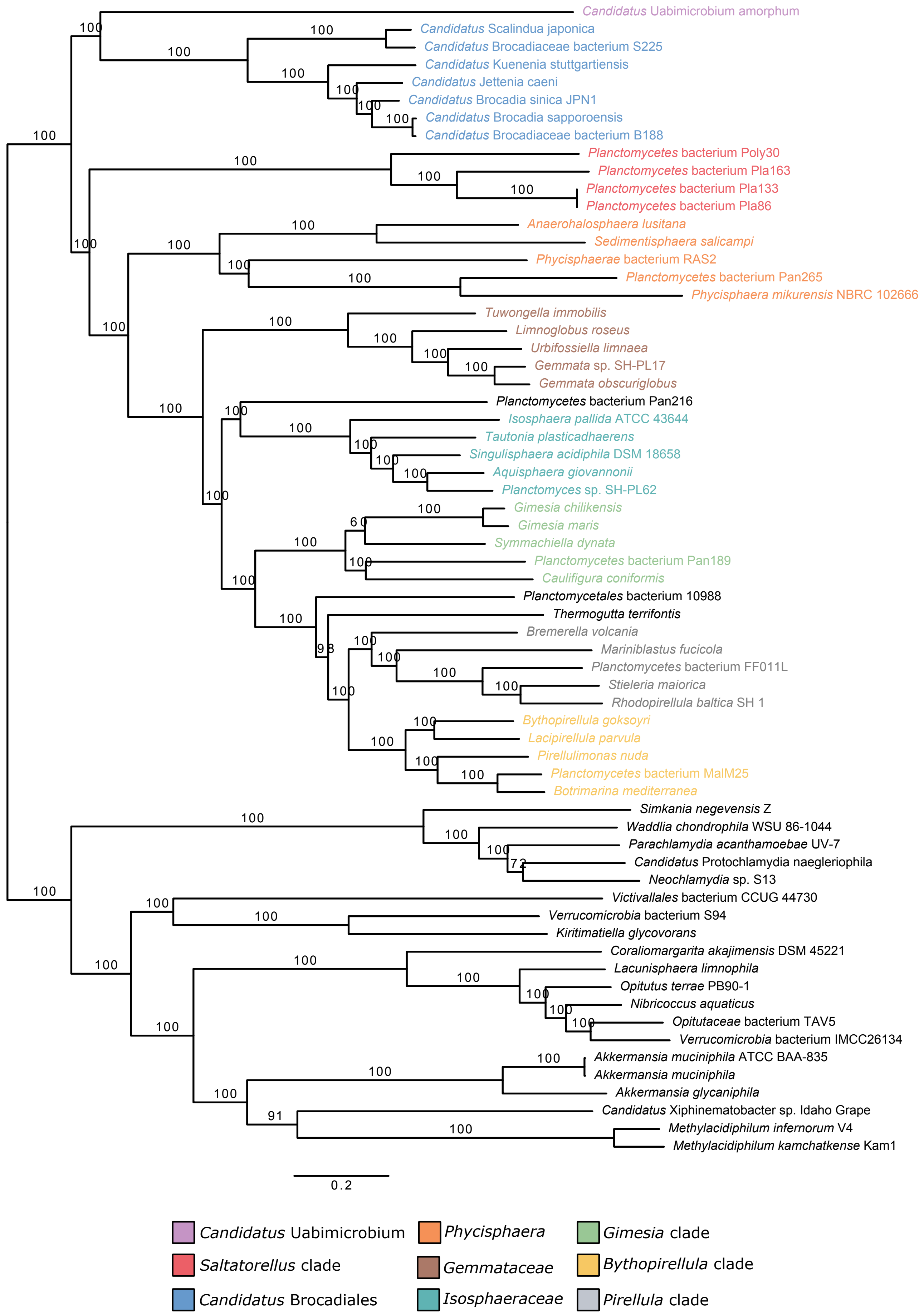

Supplementary Fig. 1c

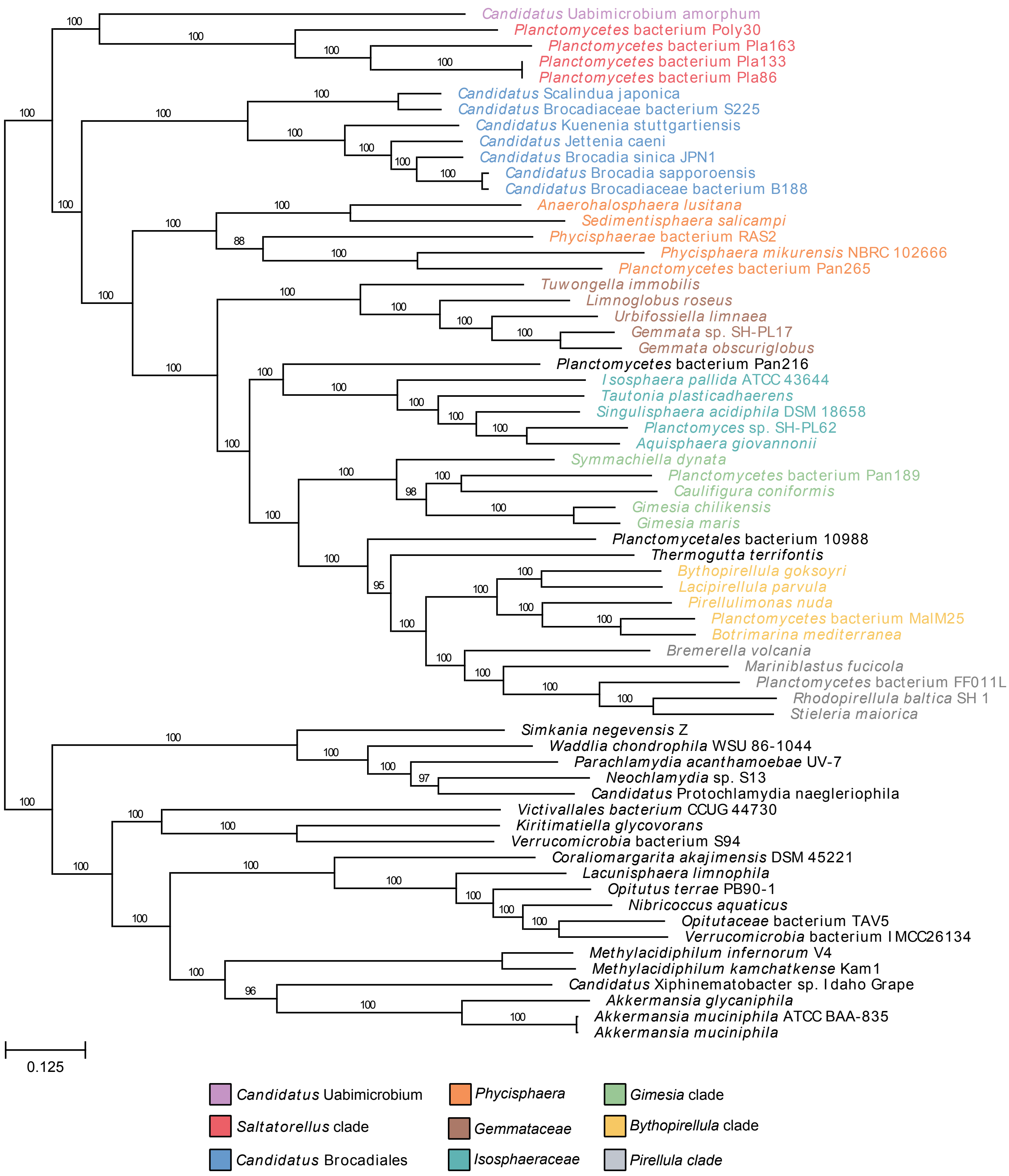

Supplementary Fig. 2a

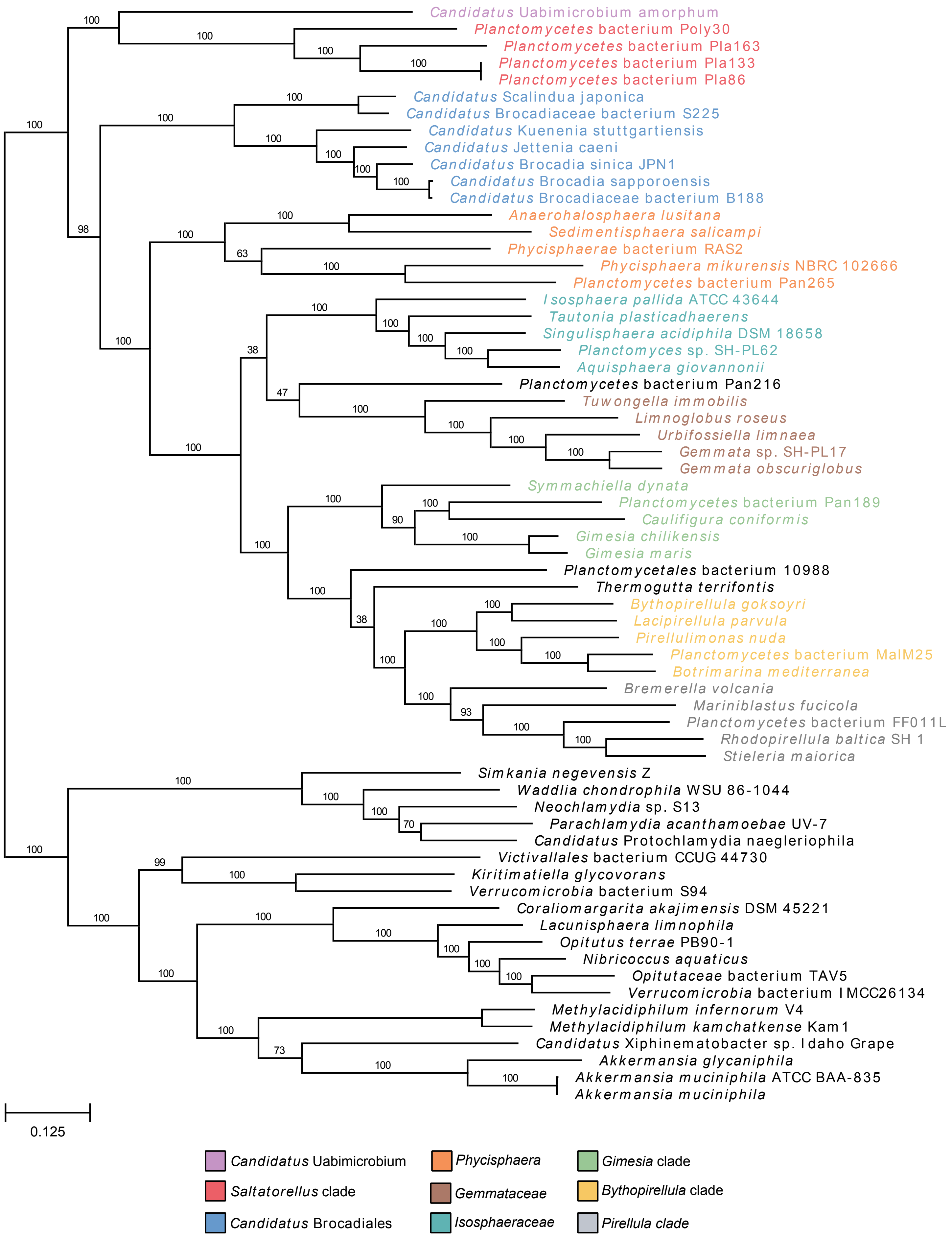

Supplementary Fig. 2b

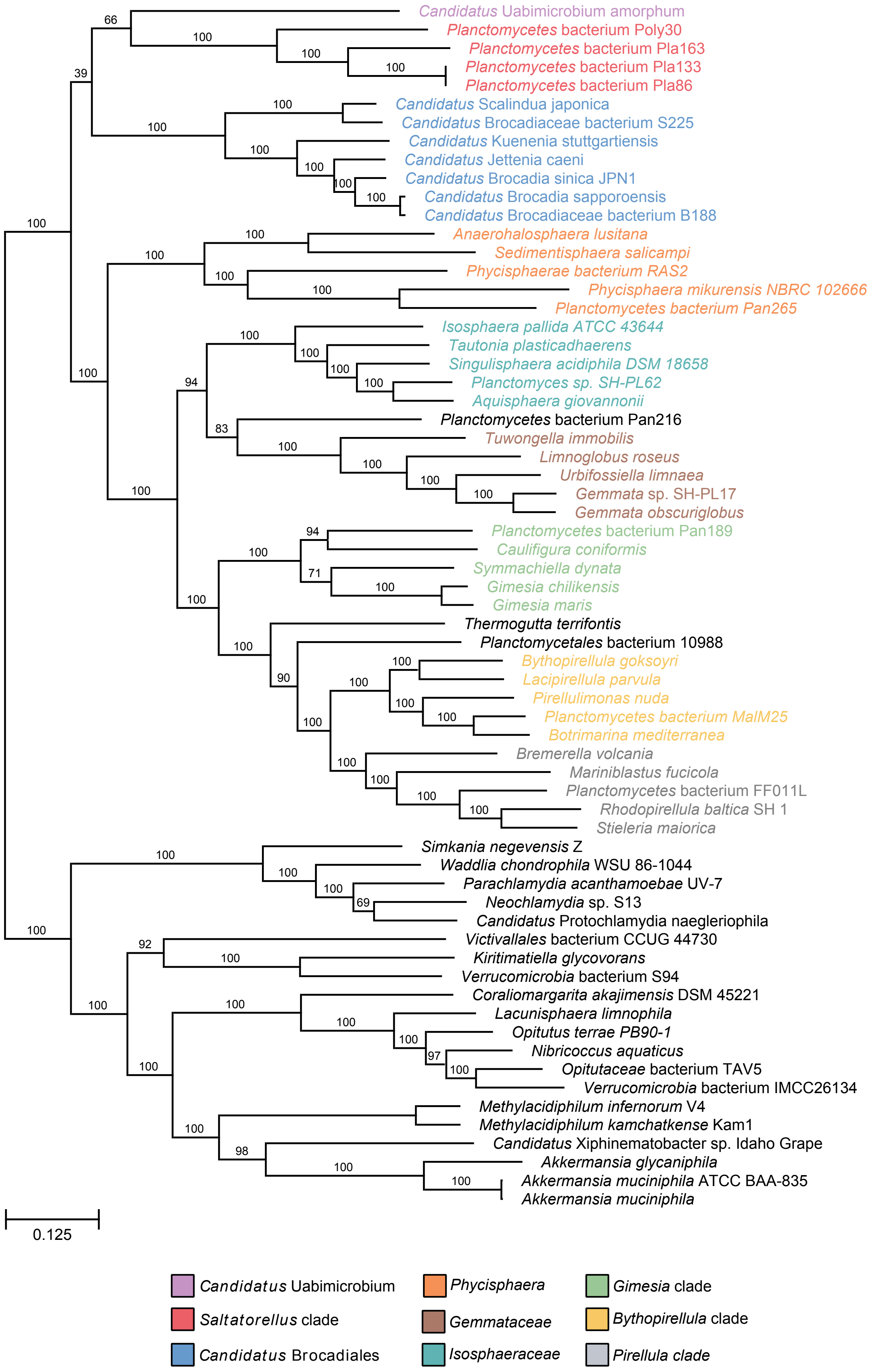

Supplementary Fig. 2c

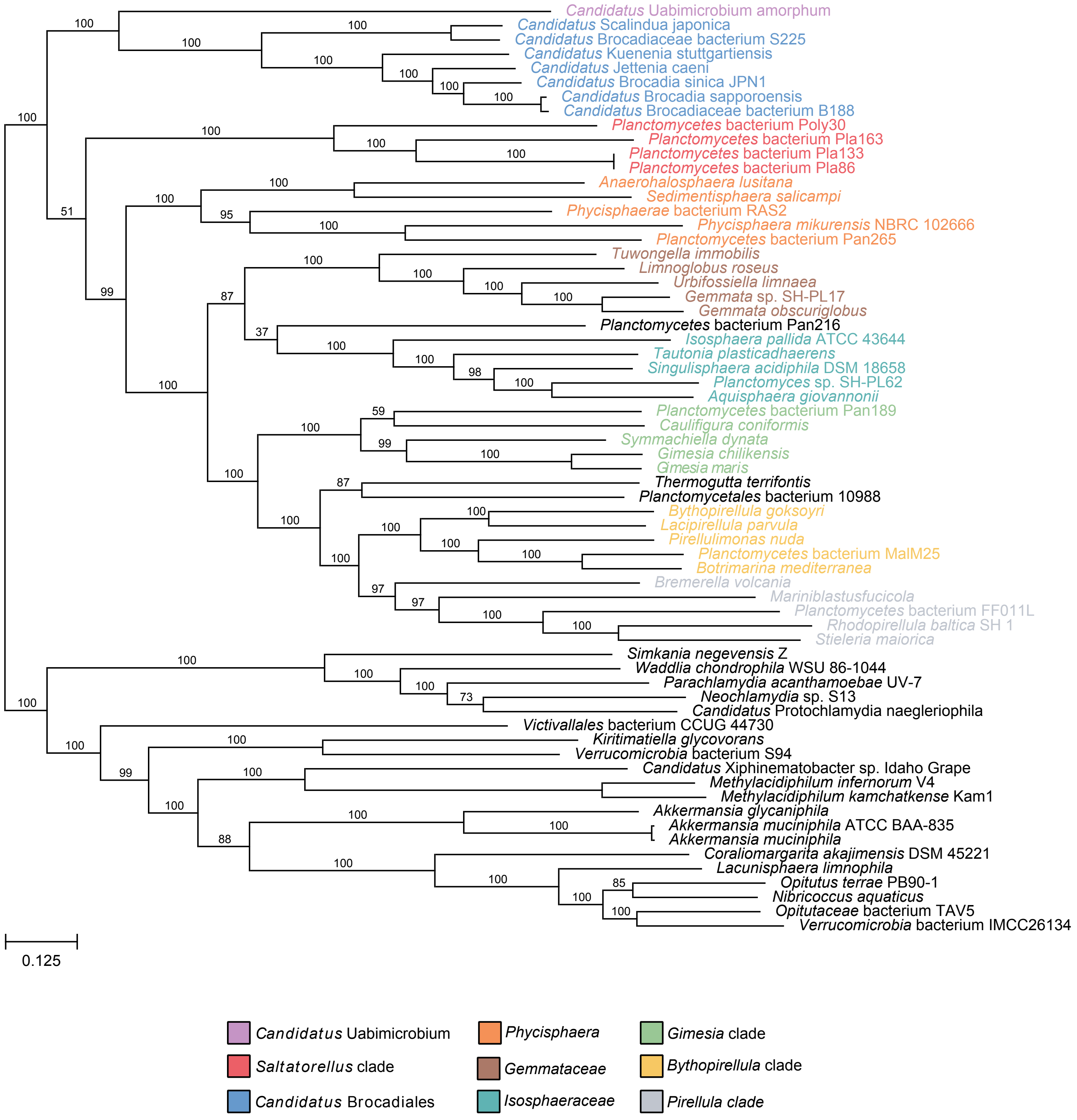

Supplementary Fig. 2d

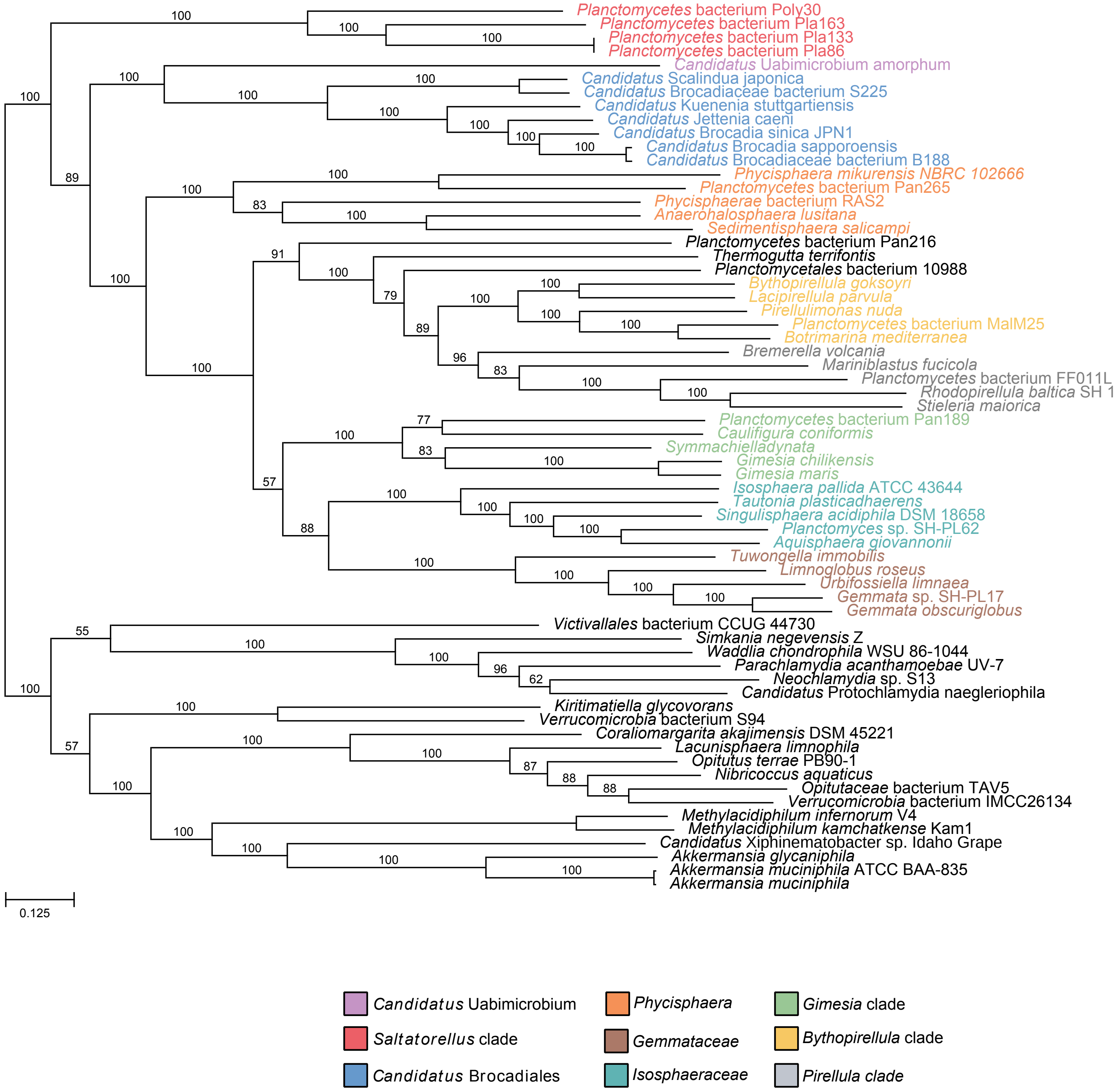

Supplementary Fig. 3

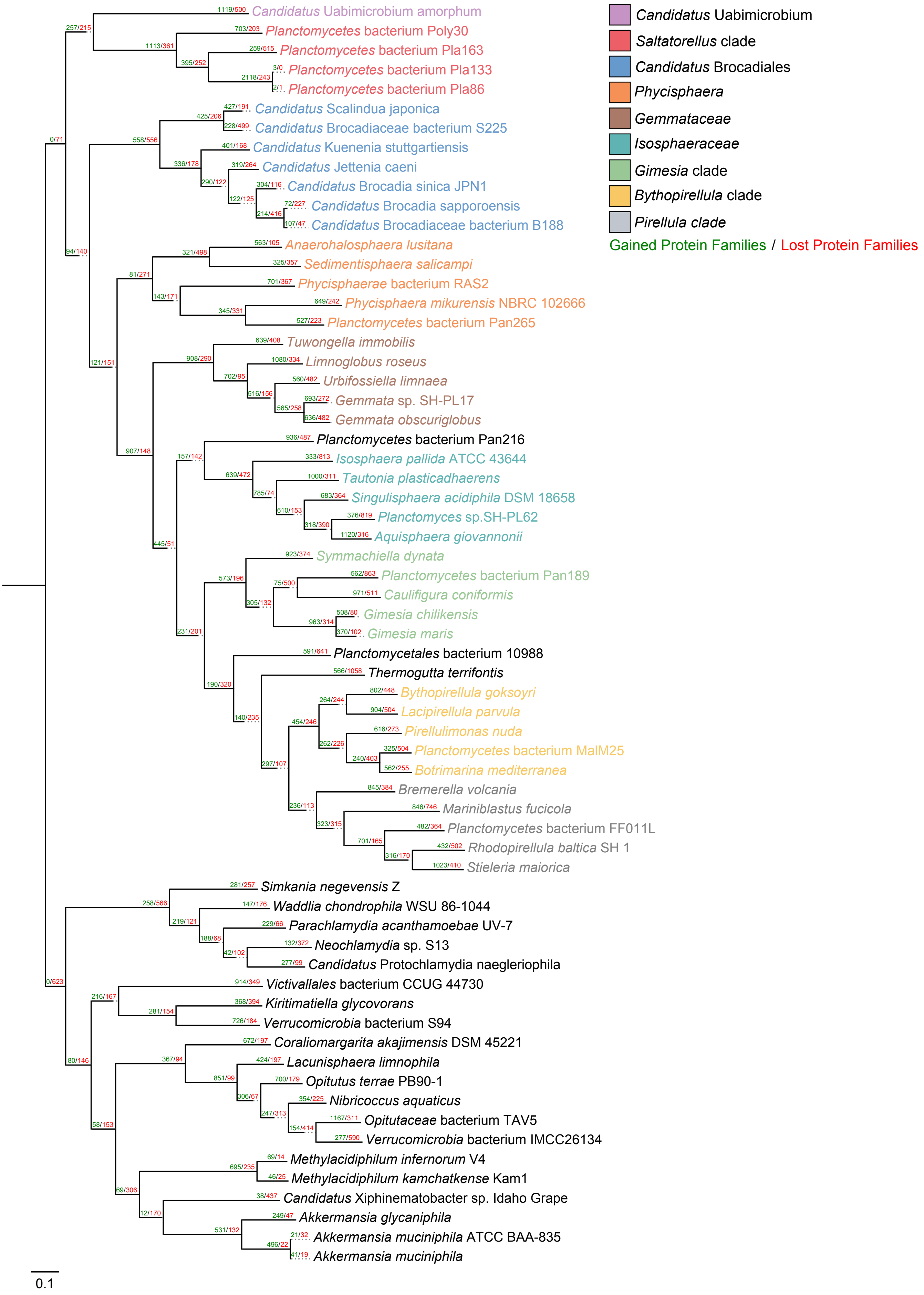

Supplementary Fig. 4

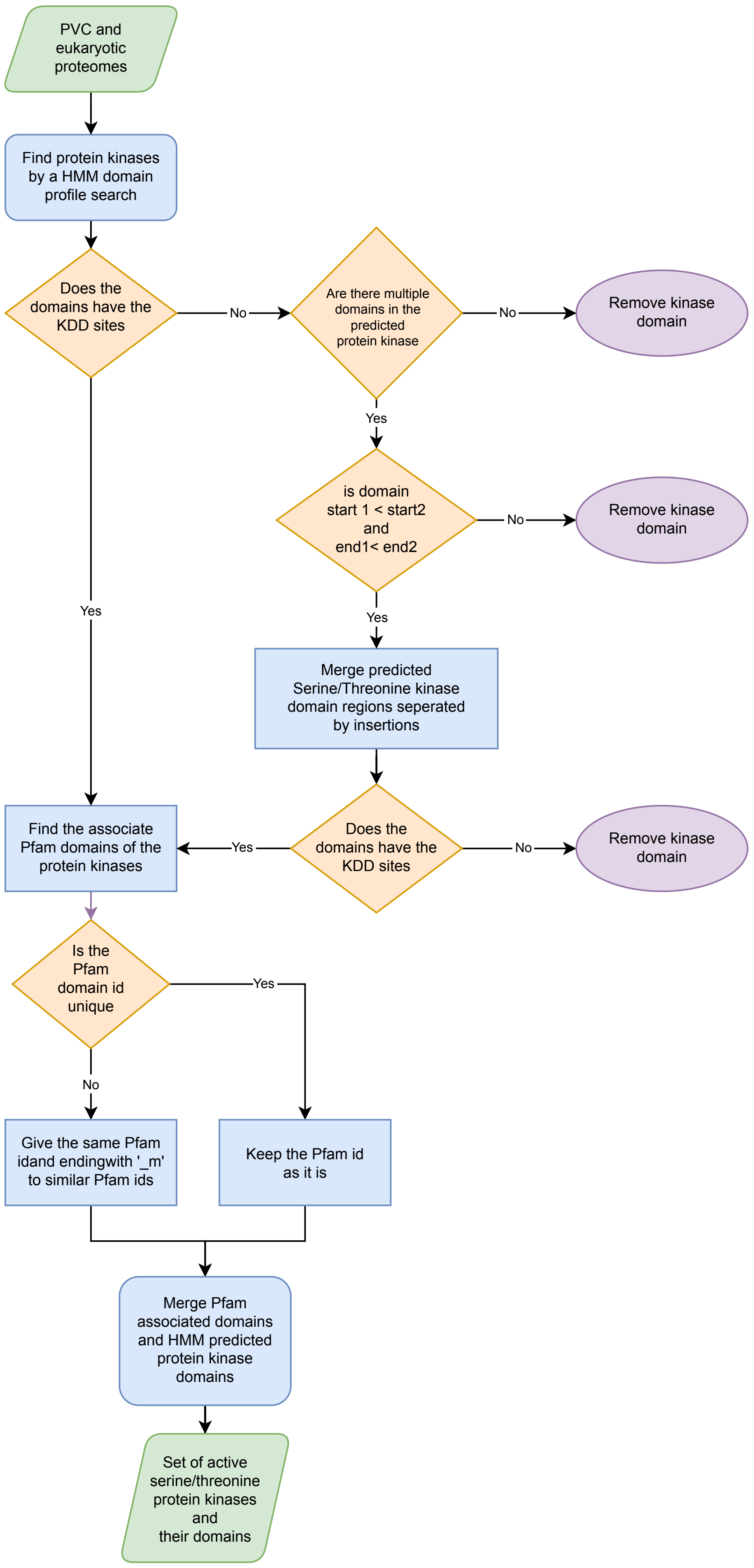

Supplementary Fig. 5

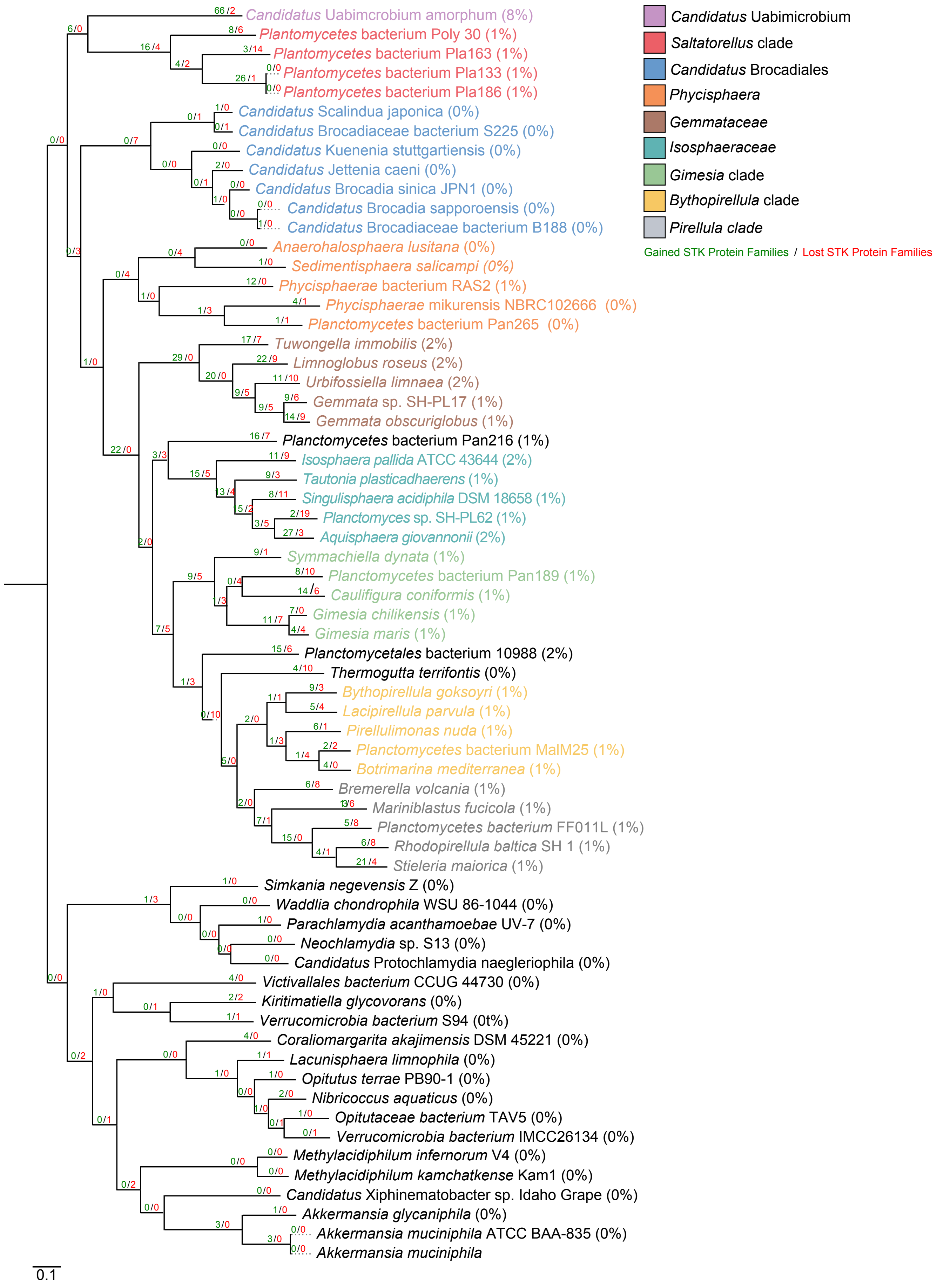

Supplementary Fig. 6

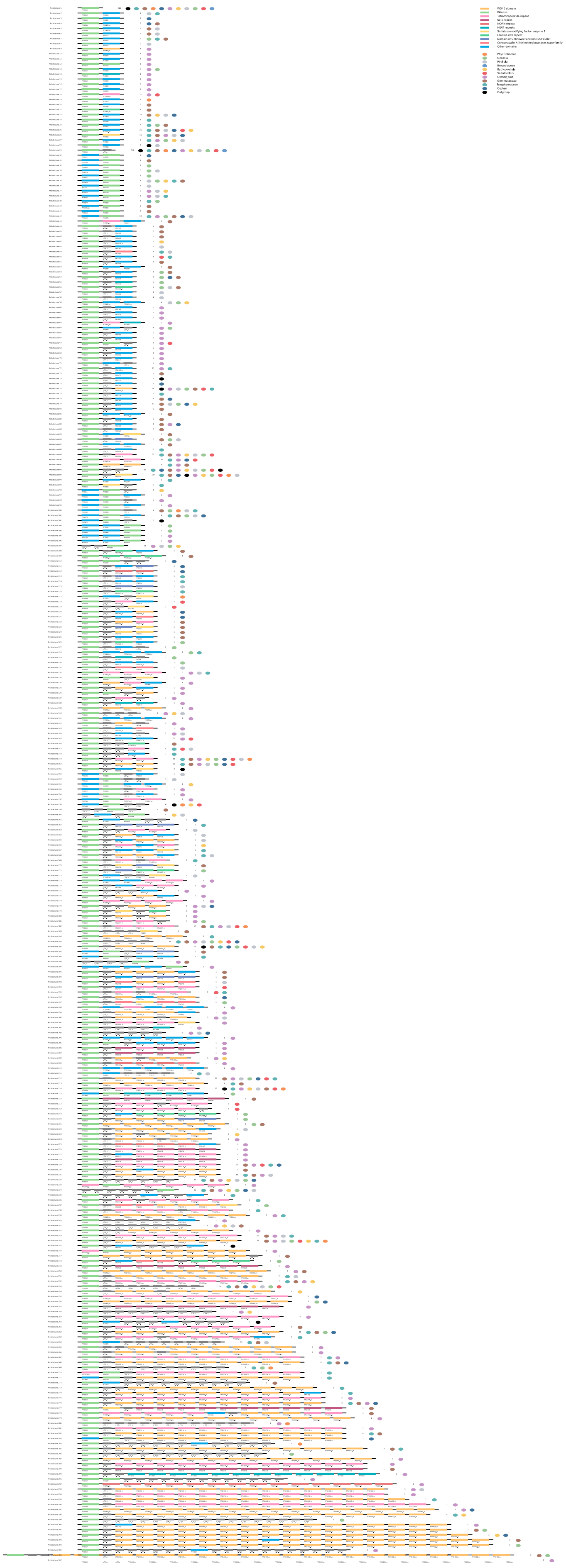

Supplementary Fig. 7

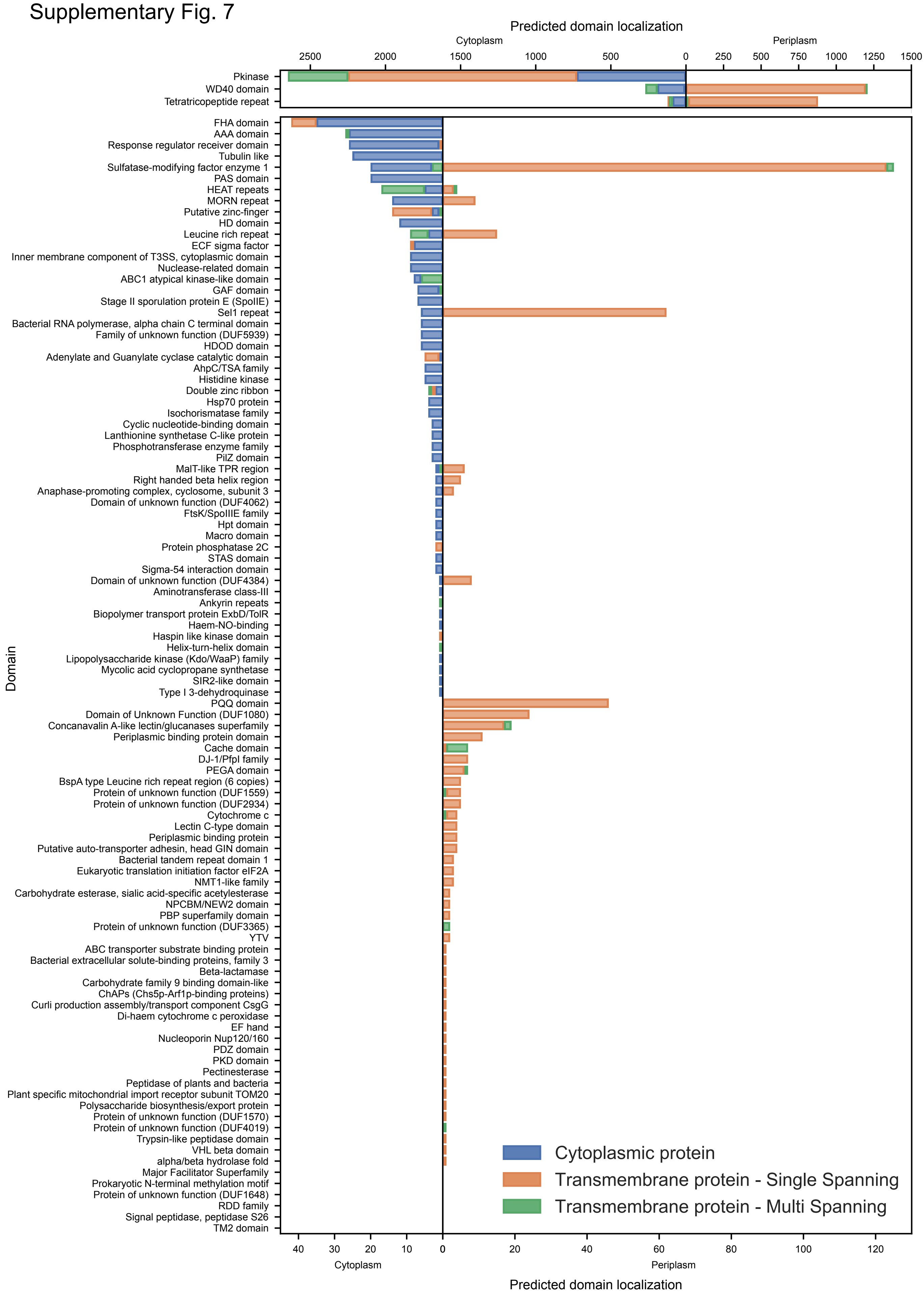

Supplementary Fig. 6

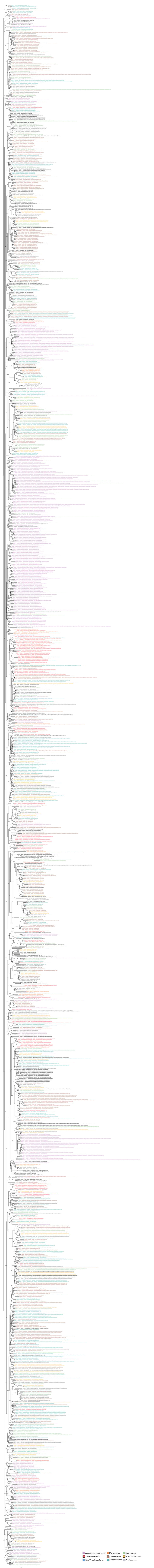

Supplementary Fig. 9

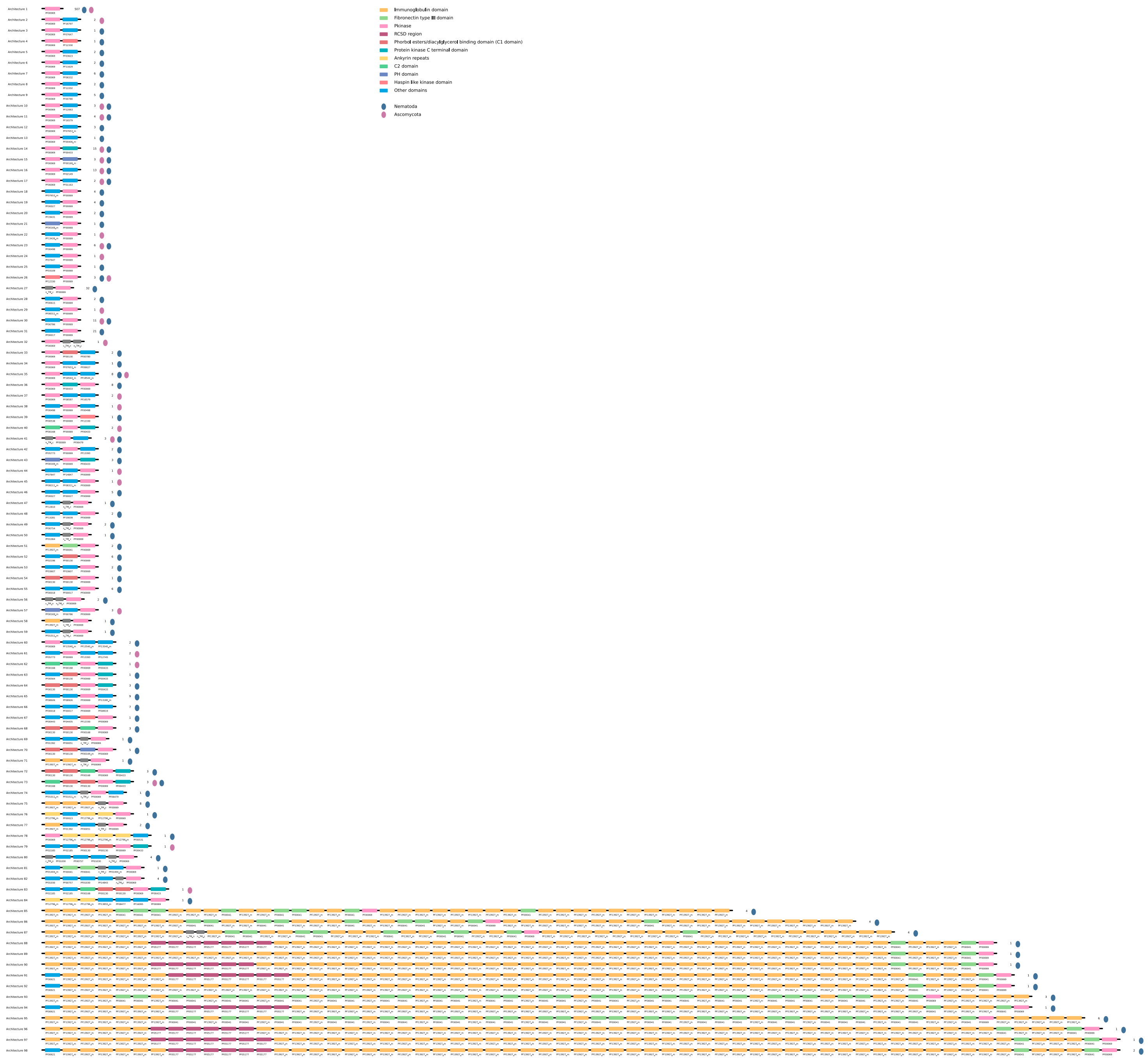

Supplement: evae068_Supplementary_Data [file evae068_supplementary_data.zip › Supplementary.Legends+Figures.pdf]
